# Repeated Intratracheal Instillation of PM10 Induces Lipid Reshaping in Lung Parenchyma and in Extra-Pulmonary Tissues

**DOI:** 10.1371/journal.pone.0106855

**Published:** 2014-09-26

**Authors:** Angela Maria Rizzo, Paola Antonia Corsetto, Francesca Farina, Gigliola Montorfano, Giuseppe Pani, Cristina Battaglia, Giulio Sancini, Paola Palestini

**Affiliations:** 1 Department of Pharmacological and Biomolecular Sciences (DiSFEB), Laboratory of Membrane Biochemistry and Applied Nutrition, Università degli Studi di Milano, Milano, Italy; 2 Department of Health Science (DISS), POLARIS Research Center, University of Milano-Bicocca, Monza, Italy; 3 Department of Medical Biotechnologies and Translational Medicine (BIOMETRA), Laboratory of Genomic Technologies Università degli Studi di Milano, Segrate, Italy; Medical University of South Carolina, United States of America

## Abstract

Adverse health effects of air pollution attributed mainly to airborne particulate matter have been well documented in the last couple of decades. Short term exposure, referring to a few hours exposure, to high ambient PM10 concentration is linked to increased hospitalization rates for cardiovascular events, typically 24 h after air pollution peaks. Particulate matter exposure is related to pulmonary and cardiovascular diseases, with increased oxidative stress and inflammatory status. Previously, we have demonstrated that repeated intratracheal instillation of PM10sum in BALB/c mice leads to respiratory tract inflammation, creating in lung a condition which could potentially evolve in a systemic toxic reaction. Additionally, plasma membrane and tissue lipids are easily affected by oxidative stress and directly correlated with inflammatory products. With this aim, in the present investigation using the same model, we analyzed the toxic potential of PM10sum exposure on lipid plasma membrane composition, lipid peroxidation and the mechanisms of cells protection in multiple organs such as lung, heart, liver and brain. Obtained results indicated that PM10 exposure led to lung lipid reshaping, in particular phospholipid and cholesterol content increases; concomitantly, the generation of oxidative stress caused lipid peroxidation. In liver we found significant changes in lipid content, mainly due to an increase of phosphatidylcholine, and in total fatty acid composition with a more pronounced level of docosahexaenoic acid; these changes were statistically correlated to lung molecular markers. Heart and brain were similarly affected; heart was significantly enriched in triglycerides in half of the PM10sum treated mice. These results demonstrated a direct involvement of PM10sum in affecting lipid metabolism and oxidative stress in peripheral tissues that might be related to the serious systemic air-pollution effects on human health.

## Introduction

Recent epidemiological studies indicated how air pollution becomes a relevant factor in the occurrence of cardiovascular diseases, demonstrating an association between both long-term and short-term air pollution exposure and cardiovascular morbidity and mortality events [1], [2]. Short-term exposure, referring to a few hours exposure, to high ambient PM10 (particles ≤10 µm in aerodynamic diameter, comprising coarse, fine and ultrafine particles) concentration is linked to higher hospitalization rates for cardiovascular events [2], typically 24 h after air pollution peaks [1].

Compelling evidences indicated that PM10 causes the most serious effects on human health because of the broad range of different toxic substances that particles contain [2], [3]. Coarse particles can contain biogenic materials, such as pollen, endotoxin and spores [4]; in particular, Gram-negative bacteria were mainly found in PM10sum, while Gram-positive bacteria were predominant in PM10win [5], thus the LPS amount was greater in PM10sum (60, 5 EU/mg) than in PM10win (20, 7 EU/mg) [6]. Also transition metals and endotoxins are potential mediators of PM10 adverse effects, causing reactive oxygen species and inflammatory mediator production [7], [8].

It has been proved that lung inflammation plays a key role in enhancing the extra-pulmonary translocation of smallest particles, as confirmed by the evidence that particle translocation is markedly increased following LPS treatment [9]. Moreover, ultrafine particles (UFPs ≤0.1 µm) are able to over-pass the lung clearance process and enter into the alveolar epithelium [10], [11], thus increase the possibility of their translocation through the alveolar blood barrier (ABB) [12], [13] and involving UFPs in cardiopulmonary diseases [14] and induction of neuroinflammation [15], [16].

Furthermore, it must be taken into account the release into bloodstream of pro-oxidative and pro-inflammatory mediators produced in lung exposed to PM; these mediators are also responsible for adverse systemic effects [3], [17], [18].

Systemic adverse effects could be induced by numerous chemical species adsorbed onto particles, such as soluble metals or polycyclic aromatic hydrocarbons (PAHs) [19], [20], [21].

Because PM-associated water-soluble metals can be leached off in the lung lining fluid, they are likely to be translocated to the pulmonary vasculature, heart, and other extra-pulmonary organs before dilution in the systemic circulation and clearance by liver [22]. Due to the large surface area, UFPs are able to absorb large amounts of organic molecules compared to larger particles. Ambient air particles demonstrated that UFPs have a higher percentage of both elemental and organic carbon as well as PAHs so contributing to the generation of reactive oxygen species and oxidative stress in macrophages and epithelial cells [23].

Consistent with these assumptions, in a previous work [24] we recently determined that repeated intratracheal instillation of PM10sum in BALB/c mice leads to the induction of inflammation in the respiratory tract, creating in lung a condition which could potentially evolve in a systemic toxic reaction. Indeed, after PM10sum intratracheal instillation, we demonstrated an increase in inflammatory and coagulation markers in blood and heart tissue, and a concomitant increase in markers of brain blood barrier (BBB) damage as well as oxidative stress in brain parenchyma [24].

Definitely, PM reach in endotoxin could trigger a local inflammatory response in the lung, allowing the translocation of smallest particles as well as of mediators across the ABB into bloodstream and then causing systemic toxicity. Moreover, it is likely that, after translocation of smallest particles, both their organic and metal components can result in systemic oxidative stress [25], [26].

A consequence of oxidative stress is membrane lipid peroxidation, primarily involving polyunsaturated fatty acids. Lipid peroxidation generates a constellation of products among which are reactive electrophiles such as epoxides and aldehydes [27], [28]. There is increasing evidence that aldehydes are involved in many pathophysiologic effects associated with oxidative stress in cells and tissues [26]. Malondialdehyde is a major product of lipid peroxidation, able to react with nucleic acid bases at physiological pH to form adducts resulting mutagenic in mammalian cells [29] and carcinogenic cells [30].

At last, a significant topic poorly investigated is the possible toxic effects of PM10 on the cell membranes; in particular, the PM10 could mediate reshaping of phospholipid pattern and their fatty acid composition. Lipids alter the geometric properties of membranes, interface between the cellular and the extracellular microenvironment being involved in the signalling process in response to exogenous stimuli. Cell membrane controls protein traffic and provides messenger molecules that mediate cell-cell communication, suggesting that advances in our understanding of lipid modifications induced by PM could better disclose PM adverse effects at a molecular level.

Using an *in vitro* model, Brandenberger et al. [31] demonstrated that particle exposure induces an increase in plasma membrane surface area which correlates with the total particle surface area that cells are exposed to. This increase may be explained by lipid trafficking to the apical plasma membrane and may be interpreted as a protective reaction of the cells against particle induced stress. In according, Beretta et al. [32] found an increase in phospholipid phosphorous in alveolar cells exposed to organic extract of tire debris, in a dose dependent manner, but minor differences in the phospholipid composition.

Starting from these considerations, in the present investigation the toxic potential of PM10sum on lipid plasma membrane composition, lipid peroxidation and the mechanisms of cell protection have been analysed in multiple organs such as lung, heart, liver and brain of PM10sum exposed BALB/c mice.

In particular, we measured in these organs lipid and fatty acid content and composition, the lipid oxidation as well as heme oxygenase-1 (HO-1) and cytochrome 1B1 (Cyp1B1) protein levels, after the last of three intratracheal instillations of PM10sum. The activation of HO-1 appears to be an endogenous defensive mechanism used by cells to reduce inflammation and tissue damage in injury models, while Cyp1B1 is involved in the activation of many xenobiotic as well as PAHs metabolism. Moreover the mRNA levels of Cyp1B1, HMOX, IL-1β, MIP2, MPO, miR-21 and miR-155 were measured as inflammation markers in lung and blood. Blood and lung parameters were correlated to heart, liver and brain lipid changes resulting in interesting findings.

## Materials and Methods

### Animals

Male BALB/c mice (7–8 weeks old) were purchased from Harlan; food and water were administered ad libitum. Mice were housed in plastic cages under controlled environmental conditions (temperature 19–21°C, humidity 40–70%, lights on 7 a.m.–7 p.m.). Animal use and care procedures were approved by the Institutional Animal Care and Use Committee of the University of Milano Bicocca and complied with guidelines set by Italian Ministry of Health (DL 116/92); invasive procedures have been performed under anaesthesia and all efforts were made to minimize suffering.

### PM sources and characterization

Atmospheric PM10sum was collected during summer 2008 in a Milan urban area as described in previous paper [33]. The chemical characterization of PM10 collected during summer 2008 doesn't differ from these of PM10 collected in summer 2006 and 2007 [6]. The procedure of particles recovering is fully described in [24].

### Intratracheal PM10sum instillation

Animal testing was carried out by intratracheally instilling 6 mice for each experimental group. Briefly, BALB/c mice were anaesthetised with a mixture of 2.5% isoflurane (Flurane) and kept under anaesthesia for all the durance of the instillation procedure. Intratracheal instillation with 100 µg of PM10sum in 100 µl of isotonic saline solution or 100 µl of isotonic saline solution (sham) has been performed by means of MicroSprayer Aerosolizer system (MicroSprayer Aerosolizer- Model IA–1C and FMJ-250 High Pressure Syringe, Penn Century, USA), as described [34], [35], [36]. The intratracheal instillation was performed on days 0, 3, and 6; 24 h after the last instillation, mice from each experimental group were euthanized with an anaesthetic mixture overdose (Tiletamine/Zolazepam-Xylazine and isoflurane). Experimental doses and time course have been established in according to [24].

### Histological analysis

Lung, Heart, Brain and Liver from sham and PM10sum-treated mice were excised and immediately formalin fixed and processed for histology as previously described [24].

Samples were qualitatively screened by means of Zeiss Axioplan microscope equiped with 40x magnification and images were acquired using Zeiss AxioCam MRc5 digital camera interfaced with the Axiovision Real 4.6 software.

### Lung, liver, brain and heart parenchyma protein analysis

Lung, liver, brain and heart of sham and PM10sum-treated mice were quickly excised and washed in ice-cold isotonic saline solution. For the detection and quantification of proteins, organs were minced at 4°C, suspended in NaCl 0.9%, briefly homogenized for 30 seconds at 11000 rpm with Ultra-Turrax T25 basic (IKA WERKE) and sonicated for 30 seconds. Samples were submitted to trichloroacetic acid (TCA) precipitation according to the procedure described by Farina et al. [36]. The pellets were suspended in water and protein quantity determined by BCA method (Sigma Aldrich, USA).

Thereafter, lung, liver, brain and heart homogenates of sham and PM10sum-treated mice were loaded on SDS-PAGE and submitted to electrophoresis, followed by Western blot, according to procedures described already described [36]. Homogenates were tested with specific antibodies for HO-1 (sc-10789, Santa Cruz) and Cyp1B1 (sc-32882, Santa Cruz). Then, blots were incubated for 1.5 h with horseradish peroxidase-conjugated anti-rabbit IgG (1∶5000, Pierce) diluted in PBS-Tween20/milk. Proteins were detected by ECL using the SuperSignal detection kit (Pierce, Rockford, IL). Immunoblot bands were analysed and the optical density (OD) quantified by KODAK (Kodak Image Station 2000R); all the data have been normalized to β-actin (A2066, 1∶1500, Sigma) and each protein in PM10-treated group has been normalized to the respective sham group.

### Lung and blood molecular analyses

Lung and blood from 5 sham and 5 PM10sum-treated mice were considered for RNA analysis. Lung were suspended in an appropriate volume of RNA Later; total RNA extraction of lung and blood was performed as previously described [24]. RNA quality was checked by microcapillary electrophoresis with 2100 BioAnalyzer (Agilent Technologies, Santa Clara, CA, USA). Total RNA integrity was assessed on the basis of the RIN (RNA Integrity Number) factor and presence of low molecular weight RNA molecules (including 5S rRNA and small RNAs) was verified. RNA samples were stored at −80°C until use.

Quantitative PCR (QPCR) reactions for microRNAs was performed by use of TaqMan MicroRNA Reverse Transcription (RT) kit (Applied Biosystems, Life Technologies, Inc. Carlsbad, CA, USA) and of specific miRNA primers provided with TaqMan microRNA assays, according to the manufacturer's protocol. Starting from 10 ng of total RNA for each assay, RT reactions were performed by means of Applied Biosystems 7900 Thermocycler machine. Quantitative microRNA expression analysis was carried out for miR-21 (Assay ID, 000397, Applied Biosystems) normalized against U6snRNA (Assay ID, 001973, Applied Biosystems) taken as endogenous control. For gene expression analysis, we performed QPCR starting from 1 µg of total RNA using the High Capacity cDNA Reverse Transcription kit (Applied Biosystems) and gene-specific primers provided with TaqMan Gene Expression Assays. Specifically, QPCR analysis were carried out for HMOX1 (Assay ID Mm00516005_m1), Cyp1B1 (Assay ID Mm00487229_m1), IL-1β (Assay ID Mm01336189_m1), MIP-2 (Assay ID Mm00436450_m1) and MPO (Assay ID Mm00447886_m1) genes. All data have been normalized versus glyceraldehyde-3-phosphate dehydrogenase (GAPDH, Assay ID Mm99999915_g1) gene taken as endogenous control. Reactions were run in triplicate on the Applied Biosystems 7900 HT Fast Real-Time PCR System machine. Delta Ct values were calculated using the SDS software version 2.3 (Applied Biosystems), by applying automatic baseline and standard threshold settings.

### Lipid analysis

Tissues were submitted to lipid extraction with three different chloroform/methanol mixtures 1∶1, 1∶2, 2∶1 (v/v) and partitioned chloroform/methanol/water, 47∶48∶1, v/v/v and then with water. The organic phase, after partitioning, was dried and resuspended in chloroform/methanol (2∶1) for the analysis of phospholipid (PL), neutral glycolipid and cholesterol amount. All solvent contained BHT as antioxidant.

Purification and quantitative analysis of membrane phospholipids and cholesterol was obtained using an HPLC-ELSD system (Jasco, Japan; Sedex SEDERE, FR) equipped with a LiChrospher Si 60 column (LiChroCART 250-4, Merck, Darmstadt, Germany).

The chromatographic separation was carried out as previously described [37]. By means of Evaporative Light Scattering Detector (ELSD) we detected and quantified separated PL species. After elution, samples were splitted in two aliquots. The ratio was 1∶9, i.e. one part to the detector and nine parts have been collected by Gilson Fraction Collector Model 201, in order to separate the different phospholipid classes for further gas chromatographic analysis (GC). After separation, each phospholipid class has been analysed for fatty acid composition by GC in following conditions.

Fatty acid methyl esters were obtained by transesterification with sodium methoxide in methanol 3.33% w/v and injected into Agilent (Agilent Technologies 6850 Series II) gas chromatograph, equipped with a flame ionization detector (FID) under the following experimental conditions: capillary column: AT Silar length 30 m, film thickness 0.25 µM. Gas carrier: helium, temperature Injector 250°C, detector 275°C, oven 50°C for 20 min, rate of 10°C min^−1^ until 200°C for 20 min.

Neutral glycolipids, Triglyceride and Cholesterol were separated by HP-TLC using silica gel plates (Merck, Darmstadt, Germany). Chromatography running and quantitative analysis was performed as previously described [38]. The delipidized pellet was used to assay protein amount [39].

### Statistical analysis

SPSS (IBM) was used as platform for statistical analysis. In particular, means and standard deviations were initially calculated and multiple variable two way ANOVA coupled to Bonferroni assay to determine differences between organ lipids and molecular markers of treated and untreated mice. Statistical differences with a *p* value below 0.05 were considered significant. Related graphs were drawn with GraphPad Prism 5. Furthermore, Pearson's correlations were estimated between lipid and molecular quantitative parameters within each organ as well as between different organs; Pearson's correlation coefficients with a *p* value below 0.05 were considered significant.

For HO-1 and Cyp1B1 western blot analyses in tissues from sham and PM10sum experimental groups, results have been expressed as mean ± standard error (s.e.). Data distribution was tested by Shapiro-Wilk test; statistical differences were tested accordingly by non-parametric U Mann-Whitney test. Statistical differences were considered to be significant at the 95% level (*p* value <0.05).

## Results

### Lung, heart, liver and brain parenchyma histology and lipid analysis

**Figure 1:**
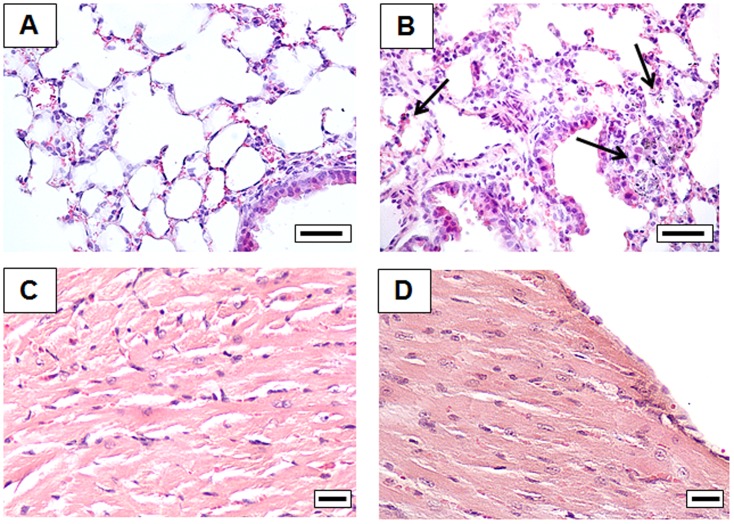
Histology of lung and heart tissue of sham (A, C) and PM10sum-treated (B, D) mice, 24 h after the third intratracheal instillation. Each figure represents the status evidenced examining 6 sham and 6 PM10sum-treated mice. A and B bars  = 50 µm. C and D bars  = 25 µm. Lung results modified from Farina et al., 2013 [24].

Lung and heart histological analyses are shown in Figure 1. Panels A and B report analyses from sham and PM10sum-treated mice lung respectively. No signs of particle accumulation signs were determined in lungs; nevertheless, it was possible to observe inflammatory cell recruitment and alveolar macrophage infiltration (arrows) in the connective surrounding terminal bronchioles and proximal alveolar sacs (panel B). Panels C and D report heart histological images; no clear sign of tissue steatosis or inflammation was evident in the heart parenchyma. Finally in brain and liver of treated mice the histological analysis did not show any difference in comparison to the organs of sham mice (data not shown); no evidence of reactive gliosis nor cortical laminar hyperintensities within the neocortex and subcortical white matters was evident in PM10sum treated mice.

**Figure 2:**
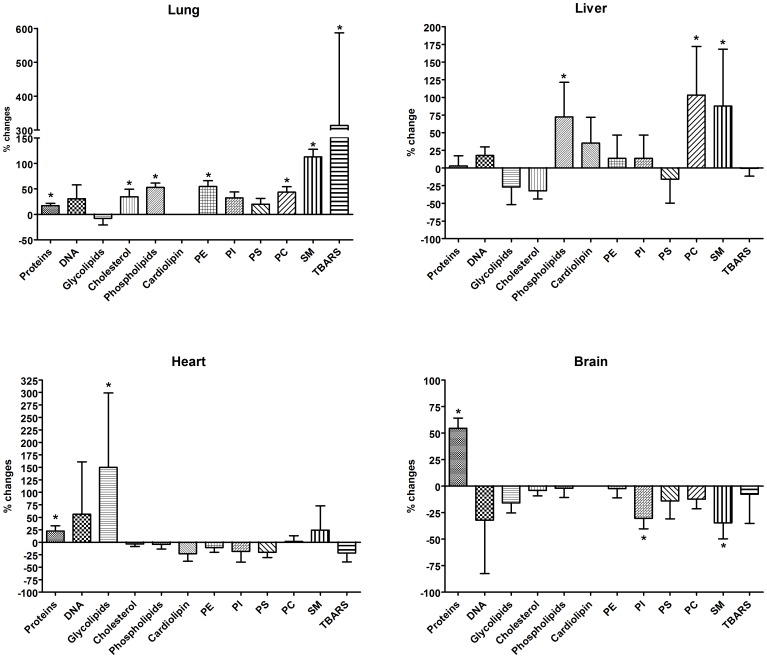
Protein, DNA, and lipid subclass changes in lung, liver, heart and brain of PM10sum-treated mice. Data were calculated as percentage variation relative to mean sham value. Statistical analysis was performed on actual amounts by a two way ANOVA coupled to Bonferroni's test. n = 6 for PM10sum and n = 5 for Sham mice. * = p<0.05. Mean data and statistical results reported in Table S1.

Data of protein, DNA, and lipid subclass amount in different tissues obtained from sham or PM10sum-treated mice are represented in Figure 2 as changes relative to mean sham. Table S1 reports the actual mean amounts of the measured parameters and the related statistical analysis, while Table S2 reports the Pearson's correlation coefficients between lipid and molecular parameters within each organ.

Obtained results showed that PM10sum exposure led to an increase in protein amount in all tissues, significantly higher in lung, brain and heart comparing to sham. As expected, the phospholipid amount significantly increased in lung after PM10sum exposure and, surprisingly, this increase was recorded also in liver. Cholesterol was increased in lung, while the quantity of neutral glycosphingolipids was significantly increased in the heart. The analysis of specific lipid classes and correlation statistical tests led to conclude that the phospholipid increase was related mainly to phosphatidylcholine (PC) in lung (r = 0.925 p<0.01), while in liver was strictly correlated with both phosphatidylethanolamine (PE) and PC (r = 0.856 and r = 0.963). Worth of note is that in lung the increase of PC was statistically correlated with cholesterol increase (r = 0.942, p<0.01).

Sphingomyelin significantly increased in lung and liver while a significant decrease was measured in brain. The tiobarbituric reactive substances (TBARS) levels were measured as lipid oxidation index. This value is significantly increased only in the lung of PM10sum-treated mice.

**Figure 3:**
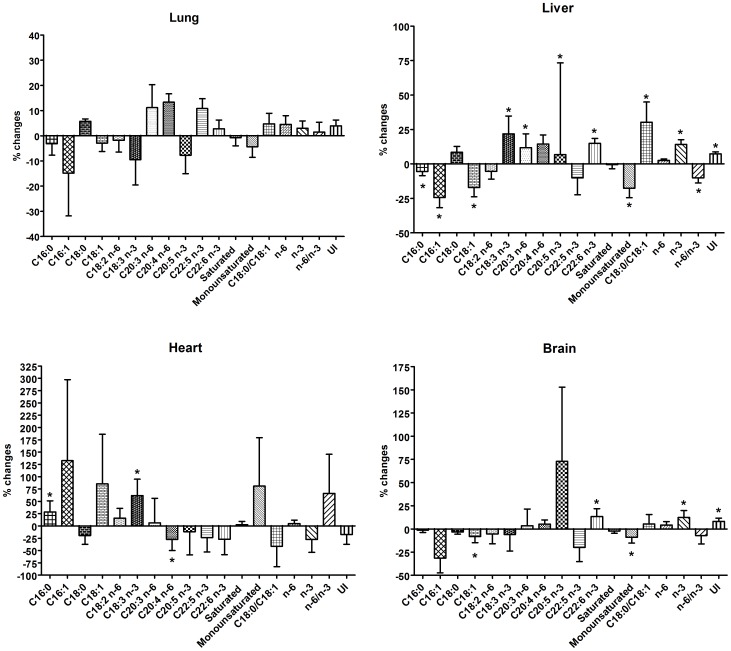
Fatty acid composition changes in lung, liver, heart and brain of PM10sum-treated mice. Data were calculated as percentage variation relative to mean sham value. U.I. =  Unsaturation Index (sum of the % unsaturated fatty acids multiplied by their number of double bonds). Statistical analysis was performed on percentage fatty acid distribution by two way ANOVA coupled to Bonferroni's test. n = 6 for PM10sum and n = 5 for Sham mice. * = p<0.05. Mean data and statistical results reported in Table S1.

In Figure 3 we reported changes relative to mean sham of total fatty acids composition in different tissues; percentage fatty acid composition and related statistical analysis are reported in Table S1. No significant differences were found in total fatty acid composition of lung from PM10sum-treated mice, comparing to sham, even if the content of C22:5 was directly correlated with TBARS (r = 0.86, p<0.05) while omega-6/omega-3 ratio was inversely correlated with it (r = −0.845, p<0.05, Table S2).

Conversely, the other tissues showed significant changes in fatty acid composition after PM10sum exposure. In particular, in both liver and brain monounsaturated fatty acids decreased, concomitantly, omega-3 and polyunsaturated fatty acids, in particular docosahexaenoic acid (DHA), increased. In liver, a significant increase of arachidonic acid (AA) was also determined.

HPLTC analysis of neutral lipids, containing cholesterol and triglycerides (TAG), underlined in 3 out of 6 PM10sum-treated mice a significantly higher amount of triglycerides (Fig. S1, Table 1) compared to sham. The level of TAG significantly correlates in heart with the fatty acid composition (Table S2). For this reason, we divided into 2 groups the PM10sum-treated mice hearts: group A with a slight increase of triglyceride amount, and group B with a high and significant increase of triglycerides. Major changes in total fatty acid composition were present in hearts from group B; in fact, due to the high TAG component, the fatty acid composition was significantly modified, with an increase of monounsaturated fatty acids and a parallel decrease of omega-3 polyunsaturated fatty acids (PUFAs), in particular DHA. Moreover, a slight decrease of AA was also measured (Table 1).

**Table 1:** Triglyceride (TAG) content and total fatty acid composition (%) of Heart from Sham and PM10sum treated mice. PM10 mice were divided into 2 groups: A) with low content of Triglycerides and B) with high content of Triglycerides.

	HEART		
Fatty Acid	Sham	PM10-A	PM10-B
C16:0	14.11±0.83	15.48±2.08	20.23±1.69*
C16:1	0.58±0.46	0.67±0.27	2.41±1.42*
C18:0	19.84±1.45	18.26±0.33*	11.78±1.45*
C18:1	7.29±0.59	8.24±2.06	23.48±4.05*
C18:2	15.45±1.54	15.57±0.91	21.41±1.62*
C20:3	0.68±0.06	0.93±0.51	0.55±0.15*
C20:4	7.86±0.45	7.21±0.77	4.34±1.02*
C20:5	0.18±0.06	0.18±0.07	0.10±0.03*
C22:5	1.53±0.19	1.55±0.14	0.79±0.25*
C22:6	32.28±2.36	31.61±0.64	14.57±5.29*
Saturated F.A.	33.95±2.04	33.74±2.33	32.01±0.71
Monounsaturated F.A.	7.87±0.87	8.91±2.06	25.88± 5.17*
C18:0/C18:1	2.74±0.31	2.34±0.59	0.52±0.15*
Omega-3 PUFAs	34.26±2.43	33.70±3.15	15.84±5.56*
Omega-6 PUFAs	23.99±1.94	23.70±1.25	26.29±0.66*
ω-6/ω-3	0.71±0.09	0.71±0.06	1.85±0.74*
U.I.	275.09±13.08	270.90±19.26	180.07±29.06**
TAG (µg/mg w.w.)	2.39±1.79	3.91±2.45	38.39±10.08**

U.I. =  Unsaturation Index (sum of the % unsaturated fatty acids multiplied by their number of double bonds).

Mean ± S.D.

*p<0.05;

**p<0.01 vs. Sham.

Fatty acid composition analysis followed HPLC separation of each phospholipid class from lipid extracts. Brain and liver results are summarized in supplemental tables (Tables S3–S4), while we focused our attention on lung and heart phospholipid fatty acid composition. Concerning lung, PE showed an increase of AA while phosphatidylserine (PS) fatty acid composition was characterized by significant changes in the major fatty acids resulting in an increased n-6/n-3 ratio (Table 2). Conversely, all the other tissues were characterized by an increase of DHA. This increase was particular evident in all the phospholipids extracted from heart, but sphingomyelin (SM) (Table 3). In brain (Table S3) PS and SM were the major affected phospholipids, while in the liver (Table S4) the increase of DHA was recorded only in PC.

**Table 2:** Phospholipid fatty acid composition of Lung from Sham and PM10sum- treated mice.

	PE		PI		PS		PC		SM	
LUNG	Sham	PM10	Sham	PM10	Sham	PM10	Sham	PM10	Sham	PM10
C16:0	22.32	20.20	19.52	15.12	12.52	12.40	52.09	57.37	**59.91**	**42.69** *
C16:1	10.29	7.30	1.83	1.48	0.53	0.63	5.32	3.44	**1.16**	**6.87** **
C18:0	15.87	13.83	23.24	24.59	**37.18**	**42.64** *	12.53	9.92	29.13	33.77
C18:1	14.12	13.91	19.96	24.00	15.57	20.26	**11.16**	**9.83** *	**3.95**	**8.54** *
C18:2	13.73	13.53	**16.90**	**24.18** *	18.13	14.47	11.00	11.83	1.95	2.62
C18:3 n-3	**2.33**	**0.87** *	**2.43**	**0.31** *	**2.48**	**0.73** *	0.45	0.28	0.51	0.40
C20:3	0.52	0.57	1.31	0.68	**1.15**	**0.94** *	0.50	0.47	1.64	2.83
C20:4	**12.68**	**18.47** *	9.28	8.12	**7.20**	**5.73** **	4.53	4.94	0.93	1.35
C20:5	0.27	0.20	0.45	0.56	0.41	0.46	0.09	0.11	0.51	0.84
C22:5	1.03	1.91	**3.15**	**0.15** *	**2.52**	**0.24** *	0.36	0.57	0.31	0.08
C22:6	6.83	9.20	1.93	0.9	2.29	1.49	**1.95**	**1.24** **	n.d.	n.d.
Saturated F.A.	38.19	34.03	42.77	39.71	**49.70**	**55.04** *	64.63	67.29	**89.04**	**76.47** *
Monounsaturated F.A.	24.42	21.21	21.78	25.48	16.10	20.90	**16.48**	**13.27** *	**5.11**	**15.41** *
Omega-3 PUFAs	10.46	12.18	**7.96**	**1.82** *	**7.70**	**2.93** **	2.85	2.20	1.33	1.58
Omega-6 PUFAs	26.93	32.58	**27.49**	**32.99** *	**26.49**	**21.14** *	16.03	17.24	4.52	6.81
n-6/n-3	2.75	2.91	**5.21**	**21.17** **	**3.52**	**7.40** **	**5.81**	**8.38** *	4.89	5.62

n.d. not detected;

*p<0.05;

**p<0.01 PM10 vs. Sham.

**Table 3:** Phospholipid fatty acid composition of Hearts from Sham and PM10sum- treated mice.

	CL		PE		PI		PS		PC		SM	
HEART	Sham	PM10	Sham	PM10	Sham	PM10	Sham	PM10	Sham	PM10	Sham	PM10
C16:0	11.07	9.79	9.35	7.71	21.15	19.23	**21.46**	**16.50** *	**32.62**	**26.44** **	**39.29**	**28.98** *
C16:1	**1.07**	**0.78** **	1.10	1.69	2.93	2.42	0.52	0.54	**0.25**	**0.10** *	1.42	1.88
C18:0	14.99	17.09	28.68	27.68	46.04	40.38	22.01	19.61	25.30	22.82	46.53	48.99
C18:1	11.63	10.65	7.30	7.06	5.09	6.47	**5.15**	**3.43** *	4.41	4.20	3.81	5.19
C18:2	50.92	48.84	**5.72**	**4.33** *	4.33	4.36	7.18	7.05	3.78	5.06	**2.46**	**4.94** *
C18:3 n-3	0.30	0.32	0.23	0.27	0.70	0.60	0.14	0.14	0.18	0.05	1.48	0.47
C20:3	1.05	0.96	0.42	0.51	0.29	0.41	0.40	0.49	0.21	0.23	0.99	0.74
C20:4	1.31	1.28	10.10	11.88	2.74	2.78	9.37	9.54	6.29	7.58	**0.87**	**2.26** *
C20:5	n.d.	n.d.	**0.90**	**0.30** *	1.21	2.39	0.10	0.36	0.03	0.04	0.30	0.36
C22:5	**0.24**	**0.44** *	**0.98**	**1.92** *	0.82	1.52	**1.94**	**2.30** *	1.38	1.42	**0.73**	**1.64** *
C22:6	**7.14**	**10.02** *	**35.07**	**42.31** *	**18.89**	**33.33** *	**31.72**	**39.80** *	**23.81**	**32.00** *	0.50	0.90
Saturated F.A.	26.06	26.88	38.04	35.39	67.18	59.84	**43.47**	**36.10** *	**63.05**	**49.26** *	85.2	77.97
Monounsaturated F.A.	13.03	11.43	8.40	8.75	8.01	8.90	**5.67**	**3.98** *	4.66	4.31	5.23	7.07
Omega-3 PUFAs	**7.63**	**10.61** *	**36.49**	**44.49** *	**21.98**	**37.87** *	**33.90**	**42.84** *	**25.70**	**33.52** *	3.56	3.19
Omega-6 PUFAS	53.28	51.08	16.23	15.75	7.37	6.87	16.95	17.08	10.28	12.91	**4.31**	**7.49** *
n-6/n-3	**7.27**	**4.89** *	0.47	0.39	0.37	0.21	0.51	0.41	0.46	0.39	2.11	2.51

n.d. not detected;

*p<0.05;

**p<0.01 PM10 vs. Sham.

### Lung, heart, liver and brain parenchyma protein analysis

**Figure 4:**
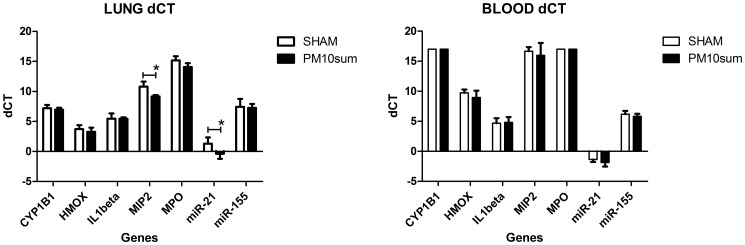
mRNA levels of Cyp1B1, HMOX, IL1β, MIP2, MPO, miR-21 and miR-155 in lung and blood. Data were normalized to housekeeping gene and expressed as delta CT; values greater or equal to 17 indicates no detectable gene expression. n = 5, * = p<0.05.

A significant increase in HO-1 level, a protein involved in inflammation and oxidative stress, was observed in lung and brain of PM10sum-treated mice, comparing to sham; on the contrary, in heart and liver HO-1 showed no significant variation (Fig. 4).

The level of Cyp1B1, a cytochrome of the P450 superfamily involved in the activation of many xenobiotic as well as in PAHs metabolism, significantly decreased in treated mice lung 24 h after PM10sum intratracheal instillation, while a significantly increase was observed in heart of treated mice, comparing to respective sham. In liver and brain, Cyp1B1 showed no significant variation (Fig. 4).

Moreover, as previously reported, many other inflammation/stress proteins were significantly increased in lung, heart, brain and inflammatory cells were present in blood and bronco alveolar lavage fluid (BALF) of PM10sum treated mice. In particular we previously recorded an increase of AMs and PMNs cells, mainly neutrophils, in BALF and blood of PM10sum treated mice [24].

### RNA expression in lung and blood: correlations with lipids

To follow inflammation markers, in both blood and lung the gene expression of different markers were measured. HO-1 (HMOX) acts as defense protein and its deficiency leads to enhanced endothelial cells injury [40]. HO-1 role is to catabolize the heme group from the cytosol, thus generating CO, biliverdin (converted to bilirubin) and Fe^2+^ thus playing a protective role against inflammation and oxidative stress [41].

One of the major markers of inflammation is TNFα, a pleiotropic cytokine [42], able to induce the release of other factors such as MIP-2 (IL-8 in humans, MIP-2 in rodents) and IL1β [43]. In mice the C-X-C chemokine macrophage inflammatory protein MIP-2 has been identified as chemoattractant of neutrophils *in vitro* and *in vivo* [44]. Interleukin-1β is a master cytokine, known to be involved in initiating the innate immune response in vertebrates and in cytokines expression [45], [46].

MPO is an inflammatory marker released by degranulation of activated neutrophils promoted by IL-1β [47].

The miR-21 is involved in negative regulation of the signaling pathway of TLR-2 [48] and TLR-4 [49], thus playing a key role in inflammatory process induced by LPS. Moschos et al. [50] and Sheedy et al. [49] hypothesized that miR-21 might be involved in the resolution rather than in the induction of inflammation.

Finally miR-155 is involved in the HMGB1/RAGE signaling pathway, whose activation lead to the enhanced expression of cytokines and molecules; thought it plays a role in particle effects, including the release of proinflammatory cytokines (such as tumor necrosis factor alpha, interleukin IL-1β, IL-6 and IL-8), adhesion molecules (i.e. vascular cell adhesion molecule 1 and intercellular adhesion molecule 1), and coagulation factors (i.e. plasminogen activator inhibitor-1, tissue-type plasminogen activator and tissue factor) [51]–[54].

**Figure 5:**
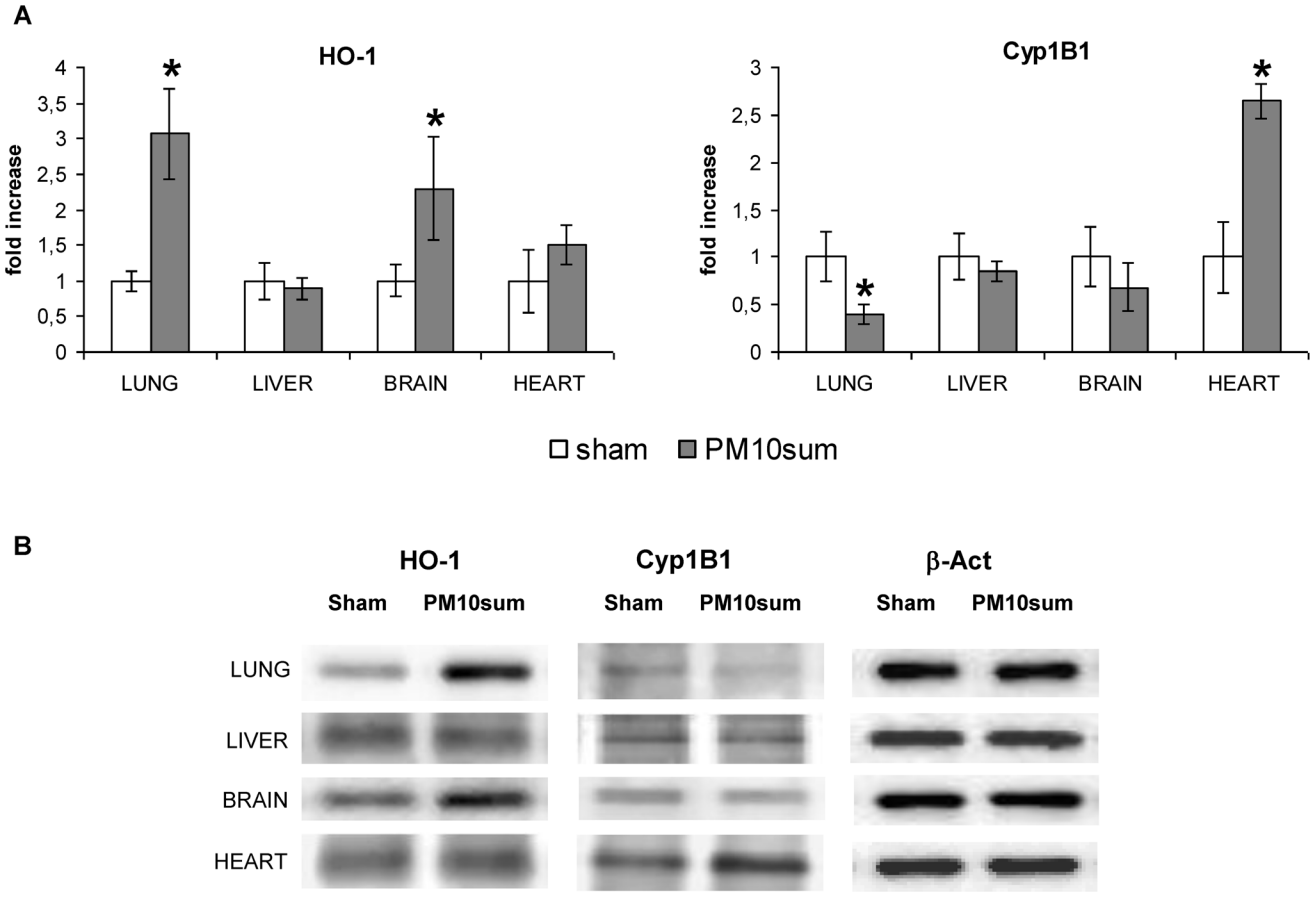
Immunoblotting analysis in lung, liver, brain and heart parenchyma from sham and PM10sum-treated mice, 24 h after the third intratracheal instillation. (A) Western blot analysis of HO-1, Cyp1B1 as optical density (OD) quantified by Kodak Image Station. The proteins have been normalized to β-actin and each protein in PM10 treated group has been normalized onto respective sham group. (B) Representative Western blotting showing HO-1, Cyp1B1 and β-actin in lung, liver, brain and heart parenchyma from sham and PM10sum-treated mice, 24 h after the third intratracheal instillation. All the data are expressed as mean ± S.E. Sham vs. PM10sum-treated: * = p<0.05.

Gene expression results are reported in Figure 5; data were normalized to housekeeping genes and reported as dCT. A significant increase of MIP2 and miR21 was recorded as inflammatory gene expression in lung, while no statistical differences were observed in blood.

**Figure 6:**
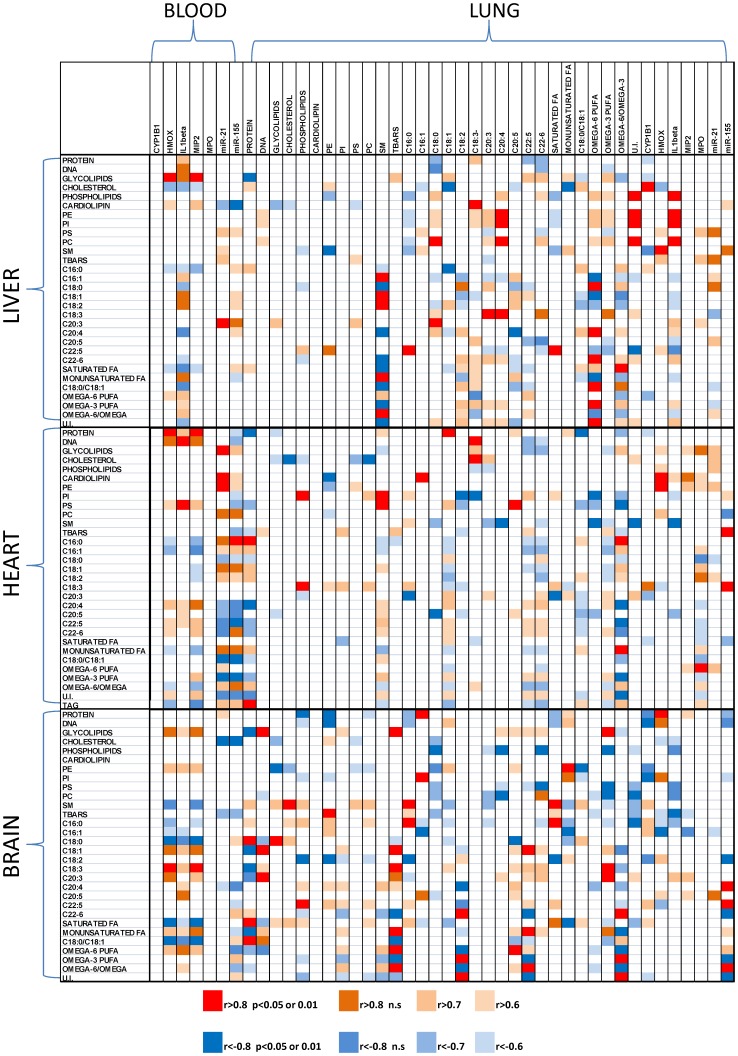
Correlation Heat Map Matrix between Blood and lung parameter couple with liver heart and brain data. Pearson's correlations were estimated using SPSS platform and transformed in a color code matrix. Only correlation with an R greater than 0.6 were coded; positive correlation are indicated with a red scale (red indicates statistical significant ones with p<0.05 or 0.01), while negative correlation are indicated with a blue scale (dark blue indicates statistical significant ones with p<0.05 or 0.01).

Correlation analysis between blood and lung molecular and lipidic parameters coupled to heart, liver and brain data revealed interesting observations. Obtained results are actually reported in Figure 6 as a simplified heath matrix.

As observable, inflammation markers measured in blood and level of proteins in lung highly correlated with tissue lipid composition in heart. Moreover, the level of AA and gene expression in lung were directly correlated with liver phospholipids.

Also brain lipids were significantly influenced by lung parameters, in particular, the levels of omega-6 and omega-3 polyunsaturated fatty acids.

## Discussion

Epidemiological and experimental studies demonstrated positive associations between adverse cardiopulmonary effects and acute and chronic exposure to concentrations of ambient air pollution currently found in major metropolitan areas [17].

The connection between exposure to PM and harmful cardiopulmonary effects has been reasonably well established, while the evidence that the central neuronal system (CNS) may be another PM target is growing. Indeed, PM might be associated with cerebrovascular and neurodegenerative diseases [55].

It is known that PM10 exposure increases plasma ET-1 levels [56], thus mediating systemic endothelial dysfunction. We demonstrated also PM10sum toxicity on cardiovascular system and brain parenchyma after PM10sum instillation, thus confirming PM systemic effects [24].

Several mechanisms have been proposed to explain the adverse health effects of particulate pollutants. These include inflammation, endotoxin effects, stimulation of capsaicin/irritant receptors, autonomic nervous system activity, pro-coagulant effects, covalent modification of cellular components and ROS production. Among these, ROS production and the generation of oxidative stress have received the most attention [57]. Ambient particles contain a large number of soluble metals including transition metals that are capable of redox cycling. Evidence is also accumulating to suggest that organic components carried on the particle surface play an important role in mediating the toxic effect. For example, PAHs can induce oxidative stress indirectly, through biotransformation by cytochrome P450 to generate redox active quinones that act as catalysts for free radical production [58].

Here, we investigated lipid variations induced in lung parenchyma after PM10sum intratracheal instillation in BALB/c mice; in parallel, we analyzed HO-1 and Cyp1b1 levels. Extra-pulmonary tissues were also affected by PM10sum exposure; we were able to measure significantly changes in lipids amount and protein levels in liver, brain and heart strictly correlated to lung and blood parameters.

### Lung

Lipid modification of lung tissue and surfactant are well described as primary effects of different particulate matter in humans and animal models [59], [60], while there are scattered data on other tissue lipid modifications.

As previously described, we found in lung the histological features and the increase of proteins and lipids related to the inflammatory process together with the increase of surfactant stored in type II cells [61]. It seems also important to notice that the increase of PC, strictly correlated with liver phospholipids, could be also related to an anti-inflammatory protection system, as some studies have demonstrated this potential for PC and its metabolites in various conditions such as oxidative stress and endotoxin induced injuries [62].

Regarding the increase of cholesterol content, this alteration is already described by electron microscopy in the lung of heavy cigarette smokers and it has been hypothesized that cholesterol may represent a degenerative change in type II pneumocytes [63].

The PM triggers pulmonary oxidative stress and inflammation by means of heterogeneous and complex mechanisms, with variable responses according to different properties of PM particles (e.g. size, charge, chemistry). Our previous work demonstrated the presence of inflammation, oxidative stress and endothelial activation in lung of PM10sum-treated mice [24]. Lung oxidative stress has been confirmed in the current work, by increase of TBARS and HO-1 levels in PM10sum-treated mice. Consequent to HO-1 increase, the lung parenchyma of PM10sum-treated mice showed a decrease in Cyp1B1 levels, due to reduced heme group bioavailability [64]. Even if the increase of TBARS indicates that lipid peroxidation might be occurred, with our protocol of exposure we did not find changes of total fatty acid composition [65]. On the contrary, when single phospholipid fatty acid composition was evaluated, we measured a significant increase of AA in PE and changes in n-6/n-3 fatty acid ratio, indicating in PI, PS and PC; the decrease of n-3 fatty acids might be related to TBARS increase in treated mice. It is known that the n-6/n-3 fatty acid ratio in alveolar cell membranes regulates pro-inflammatory cytokine release, as n-3 PUFAs are regarded to be anti-inflammatory while n-6 PUFAs, and particularly AA, are pro-inflammatory [66].

Moreover, Kampfrath [67] recently reported that chronic exposure to ambient air-borne PM2.5 increases oxidized phospholipid derivatives of 1-palmitoyl-2-arachidonyl-sn-glycero-3-phosphoryl-choline in broncoalveolar lavage fluid.

All these results, both lipids and proteins, confirmed the existence of inflammatory status in the lung of PM10sum treated mice.

### Liver

Liver is the major organ responsible for the detoxification of chemical compounds and lipid metabolism. It has been suggested that PM could reach the liver but the hepatic effects would not necessarily depend on changes in the lung or elsewhere. Instead, hepatic effects would arise from the direct contact of PM and resident phagocytic hepatic cells (e.g., Kupffer cells), which are demonstrated in *in vivo* exposure experiments [68]. Analyses of HO-1 and Cyp1B1 expression showed no modification in liver of PM10sum-treated mice comparing to sham.

Surprisingly, in liver we found significant changes in lipid content mainly due to an increase of PC and total fatty acid composition with a more pronounced level of unsaturated fatty acids, in particular DHA, and an increase of unsaturation index.

The changes of lipids are statistically correlated to molecular markers expressed in lung such as IL-1β and CYP1B1. These results might also be related with the presence in PM10sum Milan particulate of 2,3,7,8-tetrachlorodibenzo-p-diioxin (TCDD), even if we did not verify its presence. TCDD in fact is able to interact with aryl hydrocarbon receptor altering hepatic lipid metabolism; previous results have demonstrated that TCDD is able to significantly increase liver PUFA content [69]. These data may represent a preliminary indication that chronic exposure might induce metabolic liver decompensation ending in nonalcoholic steatohepatitis (NASH) and insulin resistance [70].

### Brain

Recent evidence links air pollution exposure to CNS pathology and disease [71], [72]. Even the mechanisms underlying brain pathology, induced by air pollution, have to be clarified, there are some evidence focused on neuroinflammation, oxidative stress, endothelial and glial activation, and cerebrovascular damage as possible pathways affecting the BBB [24], [73], [74].

In the present work, higher HO-1 levels were found in brain of treated mice, confirming oxidative stress induced by PM10sum. The brain is believed to be particularly vulnerable to oxidative stress, in particular neurons, as it contains high concentrations of PUFAs which are susceptible to lipid peroxidation, consumes relatively large amounts of oxygen for energy production, and has lower antioxidant defences compared to other organs [75]. Interestingly, microglia consistently generate ROS when activated by multiple pro-inflammatory triggers, such as particles [76], [77]. Oxidative stress is a common characteristic shared across numerous neurodegenerative diseases [78]–[82].

Guo has demonstrated in rats that PM10 induce also brain inflammation, and our data seem to confirm this hypothesis even if an increase of n-3 PUFAs, in particular DHA, might indicate that the organism is fighting inflammation producing pro-resolution mediators [55]. In fact, the increase of omega-3 PUFAs in brain is directly correlated with the omega-6/omega-3 ratio in lung.

Furthermore, these data are in agreement with liver results, indicating that liver PUFAs might be transported to other tissue as a refurbishment against tissue damage and inflammation. Eicosapentaenoic acid and DHA, n-3 fatty acids, are metabolized to resolvins and protectins, such as neuroprotectin D1, which has important roles in resolution of inflammation [83].

### Heart

During the last decade, further evidence in this direction has progressively accumulated, so the updated AHA-2010 scientific statement specifically defined PM exposure as “a modifiable factor that contributes to cardiovascular morbidity and mortality” [84]. Indeed, it has been shown that short-term (from few hours to weeks) exposure to PM triggers both fatal and non-fatal cardiovascular events, while long-term exposure to the same particles is associated with a greater reduction of life expectancy [85], [86].

Heart parenchyma of PM10sum-treated mice showed an increase in Cyp1B1 levels as well as endothelial activation, but no oxidative stress [24]. These results have been confirmed in the present work: the increase in Cyp1B1 levels, which happens as HO-1 (not significantly increased) does not consume the heme pool, suggests a direct translocation of the PAHs component of PM, or of smallest particles themselves, from lung to heart, possibly through the pulmonary circulation.

Nevertheless, our data indicate that PM10sum effects lipid composition in the heart and these changes are strictly correlated to molecular marker expression in blood. It has been suggested that myocardial lipid deposition, changes in lipidomic profile and possible mitochondrial impairment as db/db models of obesity might seriously impact cardiac function [86]. In particular, we found, after PM10sum treatment, an increase of triglyceride amount in 50% of mice after PM10sum treatment. The storage of triglyceride droplets within cardiomiocytes has been already described [87]; moreover, abnormal cytoplasmatic structures have been noticed in cardiac muscle cells of mice exposed to air pollution, a modification rarely noted in the sham group [88]. However, microscopy analyses did not reveal in heart parenchyma of group B PM10sum-treated mice any signs of tissue steatosis. Heart tissues of the group B were also characterized by a significantly modified total fatty acid composition, with an increase of monounsaturated fatty acids parallel, concomitantly to a decrease of omega-3 PUFAs, in particular DHA, and a consequent increase in n-6/n-3 ratio, that might be related to triglyceride accumulation.

On the contrary, as in liver and brain, heart phospholipid fatty acids were characterized by an increase of DHA also in cardiolipin. The ability of circulating DHA to displace linolenic acid from cardiolipin was already described [89]. The consequence of these changes might be related to mitochondrial respiration decrease and an increase of oxidative stress susceptibility [89]. Despite no oxidative stress was detectable in this experimental time window in heart parenchyma of our PM10sum-treated mice, the significant increase in Cyp1B1 levels could represent the first step for the upcoming start of oxidative stress and inflammation, suggested also by n-6/n-3 ratio.

## Conclusions

The association of air pollution with a number of adverse respiratory and cardiovascular health effects has been well documented. In fact, the impact of air pollutants on the respiratory system has been widely and consistently reported in last years and new epidemiological evidence which links air pollution to mortality from lung cancer is robust [90].The general consensus indicates that one of the mechanisms of air pollution-induced health effects is the oxidative stress whose main target are the lipids. By the way, membrane fatty acid composition was recently correlated to longevity [91].

Our results demonstrate that repeated exposure of PM10sum in BALB/c mice led to lung lipid reshaping, in particular an increase in phospholipid and cholesterol content; concomitantly, the generation of oxidative stress causes lipid peroxidation. The translocation of mediators, cytokines, UFPs, LPS and/or metals associated to PM10sum from lung to bloodstream might trigger systemic adverse effects, involving heart, brain and liver that we report.

In conclusion, recent epidemiological and animal toxicology studies have raised concerns about the potential impact of air pollution on peripheral tissues; in this contest, our results contribute to this vision demonstrating a direct involvement of PM10sum in affecting lipid metabolism in peripheral tissues.

## Supporting Information

Figure S1 HPTLC separation of neutral lipids extracted from Sham and PM10sum-treated mice hearts. Standards (ST) were run in 3 different concentrations to perform quantitative analysis.
(TIF)

Table S1 Content of protein, DNA, lipids, TBARS and percentage fatty acid composition in different tissue from Sham and PM10 treated mice. The table reports statistically significative differences between PM10 and Sham according to Bonfferoni test.
(DOCX)

Table S2 Pearson's correlation matrixes between data from lung, liver, heart and brain of PM10sum treated mice. Yellow underline statistically significative differences (with p<0.05 or 0.01) indicating either a positive or a negative correlations between parameters within the same tissue.
(PDF)

Table S3 Phospholipid fatty acid composition of Brain from Sham and PM10sum treated mice.
(DOCX)

Table S4 Phospholipid fatty acid composition of Liver from Sham and PM10sum treated mice.
(DOCX)
