# Supplementary figures and images for: Repeated Intratracheal Instillation of PM10 Induces Lipid Reshaping in Lung Parenchyma and in Extra-Pulmonary Tissues
Source: PLoS One. 2014 Sep 26;9(9):e106855. doi: 10.1371/journal.pone.0106855 (PMC4178018; doi:10.1371/journal.pone.0106855)

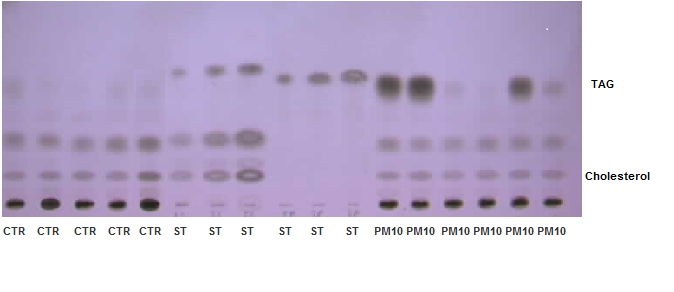

Supplement: Figure S1 — HPTLC separation of neutral lipids extracted from Sham and PM10sum-treated mice hearts. Standards (ST) were run in 3 different concentrations to perform quantitative analysis. (TIF) [file pone.0106855.s001.tif]
