# Supplementary material for: Repeated Intratracheal Instillation of PM10 Induces Lipid Reshaping in Lung Parenchyma and in Extra-Pulmonary Tissues
Source: PLoS One. 2014 Sep 26;9(9):e106855. doi: 10.1371/journal.pone.0106855 (PMC4178018; doi:10.1371/journal.pone.0106855)
Supplement: Table S1 — Content of protein, DNA, lipids, TBARS and percentage fatty acid composition in different tissue from Sham and PM10 treated mice. The table reports statistically significative differences between PM10 and Sham according to Bonfferoni test. (DOCX) [file pone.0106855.s002.docx]

**Supplemental Table S1**: Content of protein, DNA, lipids, TBARS and % fatty acid composition in different tissue form Sham and PM10 treated mice. The table reports statistical analysis significance between PM10 and Sham according to Bonfferoni test.

| **LUNG** | | | | | | | |
| --- | --- | --- | --- | --- | --- | --- | --- |
| **Dependent Variable** | **Treatment** | **Mean** | **Std. Error** | **Significance** | **95% Confidence Interval** | |  |
|  |  |  |  |  | **Lower Bound** | **Upper Bound** |  |
| Proteins μg /mg tissue | Sham | 62.78 | 3.35 | 0.04 | 55.20 | 70.36 |  |
|  | PM10 | 73.59 | 3.06 |  | 66.67 | 80.51 |  |
| DNA μg/mg tissue | Sham | 1.98 | 0.47 | 0.37 | 0.91 | 3.04 |  |
|  | PM10 | 2.58 | 0.43 |  | 1.61 | 3.55 |  |
| Glycolipids μg /mg tissue | Sham | 0.81 | 0.11 | 0.68 | 0.56 | 1.06 |  |
|  | PM10 | 0.74 | 0.10 |  | 0.52 | 0.97 |  |
| Cholesterol μg/mg tissue | Sham | 5.29 | 0.72 | 0.09 | 3.66 | 6.91 |  |
|  | PM10 | 7.11 | 0.66 |  | 5.63 | 8.59 |  |
| Phospholipids μg /mg tissue | Sham | 88.66 | 10.09 | 0.01 | 65.84 | 111.48 |  |
|  | PM10 | 135.49 | 9.21 |  | 114.66 | 156.33 |  |
| Cardiolipin μg /mg tissue | Sham | n.d. |  |  |  |  |  |
|  | PM10 | n.d. |  |  |  |  |  |
| PE μg/mg tissue | Sham | 26.74 | 3.65 | 0.02 | 18.50 | 34.99 |  |
|  | PM10 | 41.39 | 3.33 |  | 33.86 | 48.91 |  |
| PI μg/mg tissue | Sham | 3.86 | 0.91 | 0.33 | 1.81 | 5.91 |  |
|  | PM10 | 5.12 | 0.83 |  | 3.25 | 6.99 |  |
| PS μg/mg tissue | Sham | 4.11 | 0.58 | 0.33 | 2.79 | 5.43 |  |
|  | PM10 | 4.93 | 0.53 |  | 3.73 | 6.14 |  |
| PC μg/mg tissue | Sham | 44.57 | 5.23 | 0.02 | 32.74 | 56.40 |  |
|  | PM10 | 64.07 | 4.77 |  | 53.27 | 74.87 |  |
| SM μg/mg tissue | Sham | 9.38 | 1.69 | 0.00 | 5.55 | 13.21 |  |
|  | PM10 | 19.99 | 1.55 |  | 16.49 | 23.49 |  |
| TBARS pmol/mg tissue | Sham | 2.09 | 1.92 | 0.03 | -2.25 | 6.43 |  |
|  | PM10 | 8.65 | 1.75 |  | 4.69 | 12.61 |  |
| C16:0 | Sham | 31.51 | 1.57 | 0.65 | 27.97 | 35.04 |  |
|  | PM10 | 30.51 | 1.43 |  | 27.28 | 33.74 |  |
| C16:1 | Sham | 2.92 | 0.59 | 0.60 | 1.58 | 4.26 |  |
|  | PM10 | 2.49 | 0.54 |  | 1.26 | 3.71 |  |
| C18:0 | Sham | 11.61 | 0.46 | 0.31 | 10.57 | 12.64 |  |
|  | PM10 | 12.27 | 0.42 |  | 11.33 | 13.22 |  |
| C18:1 | Sham | 21.11 | 1.43 | 0.76 | 17.88 | 24.34 |  |
|  | PM10 | 20.49 | 1.30 |  | 17.54 | 23.44 |  |
| C18:2 n-6 | Sham | 15.09 | 0.90 | 0.83 | 13.05 | 17.13 |  |
|  | PM10 | 14.83 | 0.82 |  | 12.97 | 16.69 |  |
| C18:3 n-3 | Sham | 0.51 | 0.08 | 0.64 | 0.34 | 0.68 |  |
|  | PM10 | 0.46 | 0.07 |  | 0.31 | 0.62 |  |
| C20:3 n-6 | Sham | 1.07 | 0.11 | 0.46 | 0.82 | 1.33 |  |
|  | PM10 | 1.19 | 0.10 |  | 0.96 | 1.43 |  |
| C20:4 n-6 | Sham | 9.94 | 0.87 | 0.29 | 7.97 | 11.90 |  |
|  | PM10 | 11.26 | 0.80 |  | 9.47 | 13.06 |  |
| C20:5 n-3 | Sham | 0.11 | 0.01 | 0.69 | 0.07 | 0.14 |  |
|  | PM10 | 0.10 | 0.01 |  | 0.07 | 0.13 |  |
| C22:5 n-3 | Sham | 1.06 | 0.09 | 0.35 | 0.86 | 1.25 |  |
|  | PM10 | 1.17 | 0.08 |  | 0.99 | 1.35 |  |
| C22:6 n-3 | Sham | 5.09 | 0.22 | 0.64 | 4.60 | 5.58 |  |
|  | PM10 | 5.23 | 0.20 |  | 4.78 | 5.68 |  |
| Total Saturated | Sham | 43.11 | 1.85 | 0.90 | 38.94 | 47.28 |  |
|  | PM10 | 42.78 | 1.68 |  | 38.97 | 46.59 |  |
| Total Monounsaturated | Sham | 24.03 | 1.88 | 0.69 | 19.79 | 28.27 |  |
|  | PM10 | 22.97 | 1.71 |  | 19.10 | 26.85 |  |
| C18:0/C18:11 | Sham | 0.58 | 0.05 | 0.71 | 0.46 | 0.69 |  |
|  | PM10 | 0.60 | 0.05 |  | 0.50 | 0.71 |  |
| Total n-6 | Sham | 26.10 | 0.81 | 0.31 | 24.26 | 27.94 |  |
|  | PM10 | 27.29 | 0.74 |  | 25.61 | 28.97 |  |
| Total n-3 | Sham | 6.76 | 0.20 | 0.48 | 6.31 | 7.21 |  |
|  | PM10 | 6.96 | 0.18 |  | 6.55 | 7.37 |  |
| n-6/n-3 | Sham | 3.87 | 0.15 | 0.78 | 3.53 | 4.22 |  |
|  | PM10 | 3.93 | 0.14 |  | 3.62 | 4.25 |  |
| UI | Sham | 135.04 | 2.68 | 0.18 | 128.97 | 141.10 |  |
|  | PM10 | 140.37 | 2.45 |  | 134.83 | 145.90 |  |

| **HEART** | | | | | | | |
| --- | --- | --- | --- | --- | --- | --- | --- |
| **Dependent Variable** | **Treatment** | **Mean** | **Std. Error** | **Significance** | **95% Confidence Interval** | |  |
|  |  |  |  |  | **Lower Bound** | **Upper Bound** |  |
| Proteins μg /mg tissue | Sham | 141.62 | 11.22 | 0.05 | 116.24 | 166.99 |  |
|  | PM10 | 173.35 | 10.24 |  | 150.19 | 196.51 |  |
| DNA μg/mg tissue | Sham | 1.10 | 0.39 | 0.28 | 0.22 | 1.99 |  |
|  | PM10 | 1.72 | 0.36 |  | 0.91 | 2.53 |  |
| Glycolipids μg /mg tissue | Sham | 0.10 | 0.05 | 0.05 | -0.02 | 0.22 |  |
|  | PM10 | 0.25 | 0.05 |  | 0.14 | 0.36 |  |
| Cholesterol μg/mg tissue | Sham | 2.14 | 0.18 | 0.76 | 1.74 | 2.54 |  |
|  | PM10 | 2.07 | 0.16 |  | 1.70 | 2.44 |  |
| Phospholipids μg /mg tissue | Sham | 42.64 | 2.01 | 0.55 | 38.11 | 47.18 |  |
|  | PM10 | 40.94 | 1.83 |  | 36.80 | 45.08 |  |
| Cardiolipin μg /mg tissue | Sham | 6.65 | 0.54 | 0.07 | 5.43 | 7.87 |  |
|  | PM10 | 5.12 | 0.49 |  | 4.01 | 6.23 |  |
| PE μg/mg tissue | Sham | 7.61 | 0.30 | 0.08 | 6.93 | 8.29 |  |
|  | PM10 | 6.80 | 0.27 |  | 6.19 | 7.42 |  |
| PI μg/mg tissue | Sham | 3.33 | 0.29 | 0.16 | 2.66 | 3.99 |  |
|  | PM10 | 2.72 | 0.27 |  | 2.11 | 3.33 |  |
| PS μg/mg tissue | Sham | 2.87 | 0.24 | 0.11 | 2.34 | 3.41 |  |
|  | PM10 | 2.30 | 0.22 |  | 1.81 | 2.79 |  |
| PC μg/mg tissue | Sham | 19.49 | 1.36 | 0.87 | 16.41 | 22.57 |  |
|  | PM10 | 19.79 | 1.24 |  | 16.98 | 22.60 |  |
| SM μg/mg tissue | Sham | 3.38 | 0.69 | 0.40 | 1.83 | 4.93 |  |
|  | PM10 | 4.21 | 0.63 |  | 2.79 | 5.62 |  |
| TBARS pmol /mg tissue | Sham | 8.80 | 0.96 | 0.18 | 6.62 | 10.98 |  |
|  | PM10 | 6.89 | 0.88 |  | 4.90 | 8.88 |  |
| C16:0 | Sham | 14.08 | 1.07 | 0.02 | 11.66 | 16.49 |  |
|  | PM10 | 18.09 | 0.97 |  | 15.89 | 20.30 |  |
| C16:1 | Sham | 0.65 | 0.36 | 0.11 | -0.17 | 1.46 |  |
|  | PM10 | 1.50 | 0.33 |  | 0.76 | 2.24 |  |
| C18:0 | Sham | 19.07 | 1.28 | 0.06 | 16.16 | 21.97 |  |
|  | PM10 | 15.32 | 1.17 |  | 12.67 | 17.97 |  |
| C18:1 | Sham | 8.40 | 2.92 | 0.10 | 1.79 | 15.01 |  |
|  | PM10 | 15.58 | 2.67 |  | 9.55 | 21.61 |  |
| C18:2 n-6 | Sham | 15.89 | 1.11 | 0.13 | 13.38 | 18.39 |  |
|  | PM10 | 18.39 | 1.01 |  | 16.10 | 20.68 |  |
| C18:3 n-3 | Sham | 0.21 | 0.03 | 0.01 | 0.15 | 0.26 |  |
|  | PM10 | 0.33 | 0.02 |  | 0.28 | 0.39 |  |
| C20:3 n-6 | Sham | 0.68 | 0.11 | 0.79 | 0.42 | 0.93 |  |
|  | PM10 | 0.72 | 0.10 |  | 0.49 | 0.95 |  |
| C20:4 n-6 | Sham | 7.81 | 0.60 | 0.03 | 6.46 | 9.17 |  |
|  | PM10 | 5.69 | 0.55 |  | 4.45 | 6.93 |  |
| C20:5 n-3 | Sham | 0.15 | 0.04 | 0.71 | 0.08 | 0.23 |  |
|  | PM10 | 0.14 | 0.03 |  | 0.06 | 0.21 |  |
| C22:5 n-3 | Sham | 1.53 | 0.16 | 0.12 | 1.18 | 1.88 |  |
|  | PM10 | 1.17 | 0.14 |  | 0.85 | 1.48 |  |
| C22:6 n-3 | Sham | 31.55 | 3.40 | 0.10 | 23.87 | 39.22 |  |
|  | PM10 | 23.08 | 3.10 |  | 16.07 | 30.09 |  |
| Total Saturated | Sham | 33.19 | 1.29 | 0.66 | 30.28 | 36.11 |  |
|  | PM10 | 34.00 | 1.18 |  | 31.34 | 36.66 |  |
| Total Monounsaturated | Sham | 9.28 | 3.11 | 0.11 | 2.25 | 16.31 |  |
|  | PM10 | 16.80 | 2.84 |  | 10.39 | 23.22 |  |
| C18:0/C18:1 | Sham | 2.44 | 0.40 | 0.10 | 1.53 | 3.34 |  |
|  | PM10 | 1.43 | 0.37 |  | 0.60 | 2.26 |  |
| Total n-6 | Sham | 22.86 | 0.68 | 0.27 | 21.31 | 24.41 |  |
|  | PM10 | 23.96 | 0.62 |  | 22.55 | 25.37 |  |
| Total n-3 | Sham | 34.67 | 3.19 | 0.06 | 27.45 | 41.89 |  |
|  | PM10 | 25.24 | 2.91 |  | 18.65 | 31.83 |  |
| n-6/n-3 | Sham | 0.67 | 0.18 | 0.10 | 0.26 | 1.07 |  |
|  | PM10 | 1.10 | 0.16 |  | 0.74 | 1.47 |  |
| UI | Sham | 272.40 | 18.43 | 0.09 | 230.70 | 314.10 |  |
|  | PM10 | 224.73 | 16.83 |  | 186.66 | 262.80 |  |

| **LIVER** | | | | | | | |
| --- | --- | --- | --- | --- | --- | --- | --- |
| **Dependent Variable** | **Treatment** | **Mean** | **Std. Error** | **Significance** | **95% Confidence Interval** | |  |
|  |  |  |  |  | **Lower Bound** | **Upper Bound** |  |
| Proteins μg /mg tissue | Sham | 169.37 | 11.23 | 0.75 | 143.97 | 194.77 |  |
|  | PM10 | 174.40 | 10.25 |  | 151.21 | 197.58 |  |
| DNA μg/mg tissue | Sham | 2.09 | 0.16 | 0.12 | 1.72 | 2.46 |  |
|  | PM10 | 2.47 | 0.15 |  | 2.13 | 2.81 |  |
| Glycolipids μg /mg tissue | Sham | 1.28 | 0.24 | 0.32 | 0.73 | 1.82 |  |
|  | PM10 | 0.93 | 0.22 |  | 0.44 | 1.43 |  |
| Cholesterol μg/mg tissue | Sham | 2.14 | 0.31 | 0.13 | 1.44 | 2.83 |  |
|  | PM10 | 1.44 | 0.28 |  | 0.81 | 2.08 |  |
| Phospholipids μg /mg tissue | Sham | 25.45 | 4.34 | 0.01 | 15.64 | 35.26 |  |
|  | PM10 | 43.87 | 3.96 |  | 34.92 | 52.83 |  |
| Cardiolipin μg /mg tissue | Sham | 0.76 | 0.13 | 0.15 | 0.47 | 1.04 |  |
|  | PM10 | 1.02 | 0.11 |  | 0.77 | 1.28 |  |
| PE μg/mg tissue | Sham | 2.79 | 0.36 | 0.45 | 1.98 | 3.59 |  |
|  | PM10 | 3.17 | 0.33 |  | 2.43 | 3.90 |  |
| PI μg/mg tissue | Sham | 2.06 | 0.26 | 0.45 | 1.47 | 2.66 |  |
|  | PM10 | 2.35 | 0.24 |  | 1.80 | 2.89 |  |
| PS μg/mg tissue | Sham | 2.44 | 0.45 | 0.54 | 1.42 | 3.47 |  |
|  | PM10 | 2.06 | 0.41 |  | 1.13 | 2.99 |  |
| PC μg/mg tissue | Sham | 14.19 | 3.40 | 0.01 | 6.51 | 21.87 |  |
|  | PM10 | 28.85 | 3.10 |  | 21.84 | 35.86 |  |
| SM μg/mg tissue | Sham | 3.97 | 1.16 | 0.05 | 1.34 | 6.59 |  |
|  | PM10 | 7.45 | 1.06 |  | 5.06 | 9.85 |  |
| TBARS pmol /mg tissue | Sham | 13.96 | 1.20 | 0.97 | 11.25 | 16.68 |  |
|  | PM10 | 13.91 | 1.10 |  | 11.43 | 16.39 |  |
| C16:0 | Sham | 21.03 | 0.38 | 0.05 | 20.19 | 21.88 |  |
|  | PM10 | 19.87 | 0.34 |  | 19.10 | 20.65 |  |
| C16:1 | Sham | 1.32 | 0.04 | 0.00 | 1.22 | 1.42 |  |
|  | PM10 | 1.00 | 0.04 |  | 0.91 | 1.09 |  |
| C18:0 | Sham | 11.65 | 0.47 | 0.15 | 10.59 | 12.71 |  |
|  | PM10 | 12.64 | 0.43 |  | 11.68 | 13.61 |  |
| C18:1 | Sham | 14.22 | 0.47 | 0.00 | 13.15 | 15.29 |  |
|  | PM10 | 11.80 | 0.43 |  | 10.82 | 12.78 |  |
| C18:2 n-6 | Sham | 22.70 | 0.56 | 0.14 | 21.43 | 23.98 |  |
|  | PM10 | 21.48 | 0.51 |  | 20.32 | 22.65 |  |
| C18:3 n-3 | Sham | 0.68 | 0.05 | 0.04 | 0.57 | 0.78 |  |
|  | PM10 | 0.82 | 0.04 |  | 0.73 | 0.92 |  |
| C20:3 n-6 | Sham | 1.17 | 0.05 | 0.05 | 1.07 | 1.27 |  |
|  | PM10 | 1.31 | 0.04 |  | 1.22 | 1.40 |  |
| C20:4 n-6 | Sham | 14.28 | 0.41 | 0.01 | 13.35 | 15.21 |  |
|  | PM10 | 16.35 | 0.38 |  | 15.50 | 17.20 |  |
| C20:5 n-3 | Sham | 0.17 | 0.04 | 0.83 | 0.08 | 0.25 |  |
|  | PM10 | 0.18 | 0.03 |  | 0.10 | 0.25 |  |
| C22:5 n-3 | Sham | 0.57 | 0.03 | 0.14 | 0.51 | 0.63 |  |
|  | PM10 | 0.52 | 0.02 |  | 0.46 | 0.57 |  |
| C22:6 n-3 | Sham | 12.21 | 0.20 | 0.00 | 11.75 | 12.67 |  |
|  | PM10 | 14.04 | 0.19 |  | 13.62 | 14.46 |  |
| Total Saturated | Sham | 32.68 | 0.80 | 0.88 | 30.88 | 34.49 |  |
|  | PM10 | 32.51 | 0.73 |  | 30.87 | 34.16 |  |
| Total Monounsaturated | Sham | 15.54 | 0.51 | 0.00 | 14.39 | 16.68 |  |
|  | PM10 | 12.79 | 0.46 |  | 11.75 | 13.84 |  |
| C18:0/C18:1 | Sham | 0.83 | 0.06 | 0.02 | 0.69 | 0.97 |  |
|  | PM10 | 1.08 | 0.06 |  | 0.95 | 1.21 |  |
| Total n-6 | Sham | 38.16 | 0.38 | 0.09 | 37.30 | 39.01 |  |
|  | PM10 | 39.14 | 0.34 |  | 38.36 | 39.91 |  |
| Total n-3 | Sham | 13.62 | 0.23 | 0.00 | 13.09 | 14.15 |  |
|  | PM10 | 15.56 | 0.21 |  | 15.08 | 16.04 |  |
| n-6/n-3 | Sham | 2.80 | 0.04 | 0.00 | 2.71 | 2.90 |  |
|  | PM10 | 2.52 | 0.04 |  | 2.43 | 2.60 |  |
| UI | Sham | 200.55 | 2.09 | 0.00 | 195.84 | 205.27 |  |
|  | PM10 | 215.25 | 1.90 |  | 210.94 | 219.55 |  |

| **BRAIN** | | | | | | | |
| --- | --- | --- | --- | --- | --- | --- | --- |
| **Dependent Variable** | **Treatment** | **Mean** | **Std. Error** | **Significance** | **95% Confidence Interval** | |  |
|  |  |  |  |  | **Lower Bound** | **Upper Bound** |  |
| Proteins μg /mg tissue | Sham | 40.56 | 2.69 | 0.00 | 34.47 | 46.65 |  |
|  | PM10 | 62.66 | 2.46 |  | 57.10 | 68.23 |  |
| DNA μg/mg tissue | Sham | 0.34 | 0.07 | 0.27 | 0.18 | 0.50 |  |
|  | PM10 | 0.23 | 0.06 |  | 0.09 | 0.37 |  |
| Glycolipids μg /mg tissue | Sham | 54.51 | 3.27 | 0.08 | 47.11 | 61.91 |  |
|  | PM10 | 45.83 | 2.99 |  | 39.07 | 52.58 |  |
| Cholesterol μg/mg tissue | Sham | 7.34 | 0.45 | 0.63 | 6.32 | 8.36 |  |
|  | PM10 | 7.04 | 0.41 |  | 6.10 | 7.97 |  |
| Phospholipids μg /mg tissue | Sham | 40.35 | 2.86 | 0.26 | 33.88 | 46.82 |  |
|  | PM10 | 35.67 | 2.61 |  | 29.76 | 41.57 |  |
| Cardiolipin μg /mg tissue | Sham | n.d. |  |  |  |  |  |
|  | PM10 | n.d. |  |  |  |  |  |
| PE μg/mg tissue | Sham | 15.87 | 1.10 | 0.81 | 13.40 | 18.35 |  |
|  | PM10 | 15.50 | 1.00 |  | 13.24 | 17.77 |  |
| PI μg/mg tissue | Sham | 1.42 | 0.14 | 0.05 | 1.11 | 1.73 |  |
|  | PM10 | 0.99 | 0.13 |  | 0.70 | 1.27 |  |
| PS μg/mg tissue | Sham | 7.75 | 0.82 | 0.35 | 5.89 | 9.61 |  |
|  | PM10 | 6.66 | 0.75 |  | 4.97 | 8.35 |  |
| PC μg/mg tissue | Sham | 11.25 | 0.94 | 0.31 | 9.12 | 13.39 |  |
|  | PM10 | 9.86 | 0.86 |  | 7.91 | 11.81 |  |
| SM μg/mg tissue | Sham | 4.05 | 0.27 | 0.00 | 3.45 | 4.66 |  |
|  | PM10 | 2.65 | 0.24 |  | 2.10 | 3.20 |  |
| TBARS pmol/mg tissue | Sham | 3.50 | 0.54 | 0.73 | 2.27 | 4.72 |  |
|  | PM10 | 3.23 | 0.50 |  | 2.11 | 4.35 |  |
| C16:0 | Sham | 21.72 | 0.20 | 0.37 | 21.27 | 22.16 |  |
|  | PM10 | 21.46 | 0.18 |  | 21.06 | 21.87 |  |
| C16:1 | Sham | 0.99 | 0.22 | 0.32 | 0.49 | 1.49 |  |
|  | PM10 | 0.68 | 0.20 |  | 0.22 | 1.13 |  |
| C18:0 | Sham | 21.60 | 0.30 | 0.12 | 20.91 | 22.28 |  |
|  | PM10 | 20.89 | 0.28 |  | 20.27 | 21.52 |  |
| C18:1 | Sham | 22.91 | 0.59 | 0.05 | 21.57 | 24.25 |  |
|  | PM10 | 21.08 | 0.54 |  | 19.85 | 22.30 |  |
| C18:2 n-6 | Sham | 0.97 | 0.10 | 0.70 | 0.75 | 1.20 |  |
|  | PM10 | 0.92 | 0.09 |  | 0.71 | 1.13 |  |
| C18:3 n-3 | Sham | 0.67 | 0.05 | 0.54 | 0.57 | 0.77 |  |
|  | PM10 | 0.63 | 0.04 |  | 0.53 | 0.72 |  |
| C20:3 n-6 | Sham | 0.87 | 0.07 | 0.74 | 0.72 | 1.02 |  |
|  | PM10 | 0.90 | 0.06 |  | 0.76 | 1.04 |  |
| C20:4 n-6 | Sham | 10.37 | 0.26 | 0.16 | 9.78 | 10.96 |  |
|  | PM10 | 10.92 | 0.24 |  | 10.38 | 11.46 |  |
| C20:5 n-3 | Sham | 0.07 | 0.02 | 0.08 | 0.03 | 0.11 |  |
|  | PM10 | 0.11 | 0.02 |  | 0.08 | 0.15 |  |
| C22:5 n-3 | Sham | 0.33 | 0.03 | 0.10 | 0.27 | 0.38 |  |
|  | PM10 | 0.26 | 0.02 |  | 0.21 | 0.31 |  |
| C22:6 n-3 | Sham | 19.52 | 0.67 | 0.02 | 18.01 | 21.03 |  |
|  | PM10 | 22.16 | 0.61 |  | 20.78 | 23.54 |  |
| Total Saturated | Sham | 43.31 | 0.43 | 0.14 | 42.34 | 44.29 |  |
|  | PM10 | 42.36 | 0.39 |  | 41.47 | 43.25 |  |
| Total Monounsaturated | Sham | 23.90 | 0.57 | 0.02 | 22.61 | 25.19 |  |
|  | PM10 | 21.75 | 0.52 |  | 20.58 | 22.93 |  |
| C18:0/C18:1 | Sham | 0.95 | 0.03 | 0.27 | 0.87 | 1.02 |  |
|  | PM10 | 1.00 | 0.03 |  | 0.93 | 1.07 |  |
| Total n-6 | Sham | 12.21 | 0.24 | 0.14 | 11.68 | 12.75 |  |
|  | PM10 | 12.73 | 0.22 |  | 12.25 | 13.22 |  |
| Total n-3 | Sham | 20.58 | 0.63 | 0.02 | 19.14 | 22.01 |  |
|  | PM10 | 23.16 | 0.58 |  | 21.85 | 24.47 |  |
| n-6/n-3 | Sham | 0.60 | 0.02 | 0.14 | 0.55 | 0.64 |  |
|  | PM10 | 0.55 | 0.02 |  | 0.51 | 0.59 |  |
| UI | Sham | 191.00 | 3.48 | 0.01 | 183.12 | 198.87 |  |
|  | PM10 | 206.65 | 3.18 |  | 199.46 | 213.84 |  |
