# Supplementary material for: Repeated Intratracheal Instillation of PM10 Induces Lipid Reshaping in Lung Parenchyma and in Extra-Pulmonary Tissues
Source: PLoS One. 2014 Sep 26;9(9):e106855. doi: 10.1371/journal.pone.0106855 (PMC4178018; doi:10.1371/journal.pone.0106855)
Supplement: Table S3 — Phospholipid fatty acid composition of Brain from Sham and PM10sum treated mice. (DOCX) [file pone.0106855.s004.docx]

**Table S3**: Phospholipid fatty acid composition of Brain from Sham and PM10sum- treated mice

|  | PE | | PI | | PS | | PC | | SM | |
| --- | --- | --- | --- | --- | --- | --- | --- | --- | --- | --- |
| **BRAIN** | **Sham** | **PM10** | **Sham** | **PM10** | **Sham** | **PM10** | **Sham** | **PM10** | **Sham** | **PM10** |
| C16:0 | 9.11 | 9.60 | **8.54** | **11.95*** | **28.05** | **18.45*** | 58.78 | 47.61 | **32.32** | **25.69**** |
| C16:1 | 1.36 | 1.11 | 1.59 | 2.95 | 1.43 | 2.59 | 1.53 | 2.16 | 2.27 | 1.30 |
| C18:0 | 27.52 | 26.01 | 45.77 | 42.05 | **22.11** | **29.72*** | 15.66 | 15.75 | **58.09** | **61.20*** |
| C18:1 | 16.03 | 16.28 | 15.89 | 12.03 | **32.96** | **22.17**** | 23.26 | 26.89 | 1.42 | 2.48 |
| C18:2 | 1.89 | 1.81 | **0.91** | **2.47*** | 1.42 | 1.89 | 1.41 | 2.93 | **0.78** | **2.15*** |
| C18:3n-3 | **0.65** | **0.50*** | 0.61 | 0.52 | 0.58 | 0.48 | 0.29 | 0.26 | 2.71 | 2.48 |
| C20:3 | 0.87 | 0.80 | **0.41** | **0.22*** | **0.27** | **0.38**** | 0.22 | 0.17 | 0.75 | 0.83 |
| C20:4 | 16.42 | 15.28 | 4.13 | 6.16 | **7.16** | **10.24*** | 2.47 | 1.56 | 0.51 | 0.99 |
| C20:5 | 0.22 | 0.32 | 0.83 | 0.77 | 0.30 | 0.31 | 1.46 | 1.17 | n.d | n.d |
| C22:5 | 0.34 | 0.52 | 0.37 | 0.66 | 0.16 | 0.29 | 0.40 | 0.53 | **0.58** | **1.06*** |
| C22:6 | 25.23 | 27.76 | 18.54 | 20.23 | **5.56** | **11.69**** | 0.92 | 0.96 | **0.59** | **1.50*** |
| Saturated F.A. | 36.63 | 35.61 | 54.30 | 54.00 | 50.15 | 48.17 | 74.44 | 63.36 | **90.41** | **86.88*** |
| Monounsaturated F.A. | 17.39 | 17.40 | 19.27 | 14.98 | **34.39** | **24.77**** | 24.69 | 29.05 | 3.69 | 3.78 |
| Omega-3 PUFAs | 26.44 | 29.09 | 19.94 | 22.17 | **6.60** | **14.55**** | 3.071 | 2.92 | **3.87** | **5.36*** |
| Omega-6 PUFAs | 19.18 | 17.89 | 5.45 | 8.85 | **8.85** | **12.50*** | 4.11 | 4.66 | **2.04** | **3.98*** |
| n-6/n-3 | 0.75 | 0.64 | 0.29 | 0.40 | **1.42** | **0.92*** | 1.34 | 1.63 | 0.55 | 0.74 |

n.d. not detected; * p<0.05; ** p<0.01 vs. Sham
