# Supplementary material for: Repeated Intratracheal Instillation of PM10 Induces Lipid Reshaping in Lung Parenchyma and in Extra-Pulmonary Tissues
Source: PLoS One. 2014 Sep 26;9(9):e106855. doi: 10.1371/journal.pone.0106855 (PMC4178018; doi:10.1371/journal.pone.0106855)
Supplement: Table S4 — Phospholipid fatty acid composition of Liver from Sham and PM10sum treated mice. (DOCX) [file pone.0106855.s005.docx]

**Table S4:** Phospholipid fatty acid composition of Liver from Sham and PM10sum treated mice

|  | PE | | PI | | PS | | PC | | SM | |
| --- | --- | --- | --- | --- | --- | --- | --- | --- | --- | --- |
| **LIVER** | **Sham** | **PM10** | **Sham** | **PM10** | **Sham** | **PM10** | **Sham** | **PM10** | **Sham** | **PM10** |
| C16:0 | 17.91 | 16.81 | 18.78 | 17.73 | 23.17 | 20.08 | **26.77** | **23.26*** | 34.96 | 42.55 |
| C16:1 | 0.63 | 0.59 | **2.64** | **0.90**** | 2.41 | 1.42 | 0.55 | 0.43 | 1.14 | 0.76 |
| C18:0 | 27.84 | 29.85 | 49.43 | 49.01 | 54.07 | 53.80 | 18.28 | 19.04 | 61.58 | 52.96 |
| C18:1 | 6.3 | 5.06 | 2.97 | 2.00 | 3.48 | 2.47 | 6.41 | 5.13 | 1.54 | 1.01 |
| C18:2 | 5.76 | 4.65 | 3.98 | 3.26 | 2.85 | 1.71 | 16.82 | 13.56 | 0.79 | 0.72 |
| C18:3 n-3 | 0.45 | 0.48 | **0.50** | **0.26*** | **0.60** | **0.32*** | 0.47 | 0.48 | n.d | n.d |
| C20:3 | 0.61 | 0.61 | 1.02 | 1.29 | 0.32 | 0.53 | 1.30 | 1.44 | n.d | n.d |
| C20:4 | 18.78 | 19.76 | 18.03 | 21.65 | 7.45 | 10.85 | 17.76 | 20.53 | n.d | n.d |
| C20:5 | 0.10 | 0.09 | n.d | n.d | n.d | n.d | 0.09 | 0.09 | n.d | n.d |
| C22:5 | 0.51 | 0.50 | n.d. | n.d | n.d | n.d | **0.28** | **0.41*** | n.d | n.d |
| C22:6 | 21.36 | 21.60 | 2.47 | 3.80 | 5.50 | 8.76 | **11.26** | **15.63**** | n.d | n.d |
| Saturated F.A. | 45.76 | 46.65 | 68.22 | 66.74 | 77.24 | 73.88 | 45.05 | 42.30 | 94.37 | 90.50 |
| Monounsaturated F.A. | 6.67 | 5.65 | **5.61** | **2.90*** | 5.89 | 3.89 | 6.97 | 5.57 | 1.28 | 1.78 |
| Omega-3 PUFAs | 22.42 | 22.67 | 3.13 | 4.15 | 6.25 | 9.15 | **12.11** | **16.60**** | n.d | n.d |
| Omega-6 PUFAs | 25.15 | 25.02 | 24.04 | 26.20 | 10.62 | 13.09 | 35.88 | 35.53 | n.d | n.d |
| n-6/n-3 | 1.16 | 1.11 | 8.78 | 8.05 | 1.91 | 1.44 | **2.98** | **2.17**** | n.d | n.d |

n.d. not detected; * p<0.05; ** p<0.01 vs. Sham
