# Supplementary material for: Repeated Intratracheal Instillation of PM10 Induces Lipid Reshaping in Lung Parenchyma and in Extra-Pulmonary Tissues
Source: PLoS One. 2014 Sep 26;9(9):e106855. doi: 10.1371/journal.pone.0106855 (PMC4178018; doi:10.1371/journal.pone.0106855)
Supplement: Table S2 — Pearson's correlation matrixes between data from lung, liver, heart and brain of PM10sum treated mice. Yellow underline statistically significative differences (with p<0.05 or 0.01) indicating either a positive or a negative correlations between parameters within the same tissue. (PDF) [file pone.0106855.s003.pdf]

Table S2: Pearson's correlation matrixes between data from lung, liver, heart and brain of PM10sum treated mice.
Yellow underline statistical differences (with p<0.05 or 0.01) indicating either a positive or a negative correlations between parameters within the same tissue
n.d.= not detected * p<0.05 ** p<0.01

LUNG	PROTEINS	DNA	GLYCOLIPIDS	CHOLESTEROL	PHOSPHOLIPIDS	CARDIOLIPIN	PE0	PI	PS	PC	SM	TBARS	C16:0	C16:0	C18:0	C18:1	C18:2	C18:3	C20:3	C20:4	C20:5	C22:5	C22:6	SATURATED FA	MONUNSATURATED FA	C18:0/C18:1	OMEGA6 PUFA	OMEGA3 PUFA	OMEGA6/OMEGA3	U.I.	CYP1B1	HMOX	IL1beta	MIP2	MPO	miR21	miR-155
PROTEINS	1	-0,619	0,664	0,504	0,129	n.d.	0,172	-0,313	0,405	0,248	-0,555	-0,804	0,407	-0,228	0,119	-0,76	0,495	-0,027	-0,361	-0,352	-0,738	-0,651	-0,669	0,422	-0,646	0,727	0,226	-0,765	0,772	-0,431	0,483	-0,175	-0,229	-0,355	0,18	0,139	-0,295
DNA	-0,619	1	-0,298	-0,273	-0,121	n.d.	-0,262	0.843*	0,026	-0,139	0,097	0,789	-0,381	0,454	0,302	0,108	-0,274	0,454	0,264	0,787	0,465	0,449	0,444	-0,362	0,3	-0,092	0,098	0,625	-0,358	0,521	-0,034	0,248	0,732	0,726	0,393	0,12	0,478
GLYCOLIPIDs	0,664	-0,298	1	0.891*	0,44	n.d.	-0,037	0,047	0.834*	0,698	-0,185	-0,269	0,351	-0,37	0,68	-0,72	0,257	-0,5	-0,328	-0,009	-0,697	0,074	-0,062	0,412	-0,688	0.827*	0,165	-0,198	0,295	-0,185	0,599	-0,073	0,136	-0,035	0,46	0,247	-0,117
CHOLESTEROL	0,504	-0,273	0.891*	1	0,781	n.d.	0,225	0,189	0.895*	0.942**	0,233	-0,015	0,657	-0,458	0,445	-0,646	-0,193	-0,691	-0,604	-0,201	-0,349	0,324	-0,14	0,704	-0,679	0,694	-0,292	-0,251	-0,069	-0,502	0,728	-0,262	-0,168	-0,053	0,585	0,488	0,215
PHOSPHOLIPIDS	0,129	-0,121	0,44	0,781	1	n.d.	0,659	0,374	0,579	0.925**	0,622	0,314	0.892*	-0,598	0,058	-0,437	-0,716	-0,634	-0,6	-0,252	0,142	0,531	-0,119	0.909*	-0,602	0,389	-0,726	-0,154	-0,515	-0,703	0,78	-0,645	-0,487	-0,223	0,348	0,376	0,726
CARDIOLIPIN	n.d.	n.d.	n.d.	n.d.	n.d.	n.d.	n.d.	n.d.	n.d.	n.d.	n.d.	n.d.	n.d.	n.d.	n.d.	n.d.	n.d.	n.d.	n.d.	n.d.	n.d.	n.d.	n.d.	n.d.	n.d.	n.d.	n.d.	n.d.	n.d.	n.d.	n.d.	n.d.	n.d.	n.d.	n.d.	n.d.	n.d.
PE	0,172	-0,262	-0,037	0,225	0,659	n.d.	1	0,128	-0,117	0,363	0,251	-0,035	0.832*	-0,725	-0,314	-0,387	-0,525	-0,113	-0,178	-0,24	0,082	0,01	-0,285	0.818*	-0,629	0,244	-0,524	-0,276	-0,235	-0,646	0,706	-0.953*	-0,646	-0,687	-0,29	-0,218	0,701
PI	-0,313	0.843*	0,047	0,189	0,374	n.d.	0,128	1	0,373	0,331	0,215	0,752	0,144	0,117	0,364	-0,326	-0,493	0,226	-0,075	0,639	0,355	0,515	0,214	0,176	-0,171	0,3	-0,167	0,371	-0,394	0,099	0,456	-0,107	0,501	0,551	0,583	0,286	0,78
PS	0,405	0,026	0.834*	0.895*	0,579	n.d.	-0,117	0,373	1	0.822*	0,169	0,137	0,394	-0,035	0,508	-0,573	-0,123	-0,489	-0,647	-0,04	-0,219	0,331	-0,145	0,442	-0,42	0,631	-0,183	-0,196	-0,014	-0,316	0,47	0,16	0,174	0,417	0.880*	0,728	0,114
PC	0,248	-0,139	0,698	0.942**	0.925**	n.d.	0,363	0,331	0.822*	1	0,518	0,25	0,748	-0,479	0,298	-0,494	-0,508	-0,741	-0,651	-0,213	-0,047	0,544	-0,067	0,784	-0,583	0,511	-0,552	-0,141	-0,378	-0,582	0,758	-0,388	-0,297	-0,023	0,605	0,568	0,503
SM	-0,555	0,097	-0,185	0,233	0,622	n.d.	0,251	0,215	0,169	0,518	1	0,673	0,394	-0,13	-0,317	0,412	-0.895*	-0,622	-0,46	-0,331	0,736	0,774	0,169	0,373	0,226	-0,444	-0.882*	0,177	-0.920**	-0,424	-0,074	-0,137	-0,531	0,127	0,2	0,492	0,528
TBARS	-0,804	0,789	-0,269	-0,015	0,314	n.d.	-0,035	0,752	0,137	0,25	0,673	1	-0,048	0,163	0,131	0,31	-0,724	-0,104	-0,014	0,438	0,72	0.860*	0,533	-0,038	0,298	-0,292	-0,417	0,653	-0.845*	0,182	-0,016	0,075	0,343	0,607	0,376	0,276	0,679
C16:0	0,407	-0,381	0,351	0,657	0.892*	n.d.	0.832*	0,144	0,394	0,748	0,394	-0,048	1	-0,616	-0,211	-0,547	-0,562	-0,433	-0,644	-0,474	0,028	0,111	-0,48	0.997**	-0,687	0,425	-0,689	-0,514	-0,213	-0.893*	0,736	-0,71	-0,676	-0,447	0,16	0,267	0,569
C16:1	-0,228	0,454	-0,37	-0,458	-0,598	n.d.	-0,725	0,117	-0,035	-0,479	-0,13	0,163	-0,616	1	-0,202	0,382	0,185	0,497	-0,108	0,092	0,386	-0,187	-0,175	-0,642	0,761	-0,405	0,169	-0,048	0,174	0,311	-0,741	0.944*	0,475	0.880*	0,501	0,542	-0,378
C18:0	0,119	0,302	0,68	0,445	0,058	n.d.	-0,314	0,364	0,508	0,298	-0,317	0,131	-0,211	-0,202	1	-0,447	0,345	-0,142	0,327	0,689	-0,583	0,314	0,567	-0,131	-0,414	0,634	0,561	0,507	0,129	0,528	0,418	0,069	0,796	0,252	0,28	-0,184	-0,001
C18:1	-0,76	0,108	-0,72	-0,646	-0,437	n.d.	-0,387	-0,326	-0,573	-0,494	0,412	0,31	-0,547	0,382	-0,447	1	-0,16	-0,052	0,254	-0,144	0,594	0,243	0,374	-0,592	0.890*	-0.971**	-0,152	0,392	-0,452	0,317	-0.895*	0,443	-0,09	0,162	-0,378	-0,102	-0,322
C18:2	0,495	-0,274	0,257	-0,193	-0,716	n.d.	-0,525	-0,493	-0,123	-0,508	-0.895*	-0,724	-0,562	0,185	0,345	-0,16	1	0,341	0,438	0,166	-0,778	-0,679	-0,034	-0,541	-0,021	0,261	0.902*	-0,114	0.874*	0,501	-0,238	0,379	0,413	-0,074	-0,216	-0,369	-0,801
C18:3	-0,027	0,454	-0,5	-0,691	-0,634	n.d.	-0,113	0,226	-0,489	-0,741	-0,622	-0,104	-0,433	0,497	-0,142	-0,052	0,341	1	0,421	0,454	0,051	-0,565	-0,19	-0,451	0,209	-0,039	0,482	-0,023	0,463	0,394	-0,246	0,251	0,811	0,421	-0,125	-0,42	0,1
C20:3	-0,361	0,264	-0,328	-0,604	-0,6	n.d.	-0,178	-0,075	-0,647	-0,651	-0,46	-0,014	-0,644	-0,108	0,327	0,254	0,438	0,421	1	0,664	-0,255	-0,075	0,67	-0,626	0,125	-0,15	0,699	0,677	0,121	0.832*	-0,173	-0,078	0,48	-0,192	-0,663	-0.879*	-0,124
C20:4	-0,352	0,787	-0,009	-0,201	-0,252	n.d.	-0,24	0,639	-0,04	-0,213	-0,331	0,438	-0,474	0,092	0,689	-0,144	0,166	0,454	0,664	1	-0,115	0,262	0,65	-0,423	-0,056	0,244	0,568	0,744	-0,027	0,778	0,181	0,037	0,875	0,374	0,067	-0,366	0,298
C20:5	-0,738	0,465	-0,697	-0,349	0,142	n.d.	0,082	0,355	-0,219	-0,047	0,736	0,72	0,028	0,386	-0,583	0,594	-0,778	0,051	-0,255	-0,115	1	0,455	0,023	-0,02	0,607	-0,704	-0,688	0,169	-0,735	-0,175	-0,354	0,131	-0,186	0,386	0,165	0,382	0,431
C22:5	-0,651	0,449	0,074	0,324	0,531	n.d.	0,01	0,515	0,331	0,544	0,774	0.860*	0,111	-0,187	0,314	0,243	-0,679	-0,565	-0,075	0,262	0,455	1	0,656	0,139	0,079	-0,154	-0,452	0,66	-0.890*	0,079	0,169	-0,085	0,241	0,512	0,408	0,304	0,805
C22-6	-0,669	0,444	-0,062	-0,14	-0,119	n.d.	-0,285	0,214	-0,145	-0,067	0,169	0,533	-0,48	-0,175	0,567	0,374	-0,034	-0,19	0,67	0,65	0,023	0,656	1	-0,44	0,177	-0,194	0,283	0.973**	-0,475	0,729	-0,18	0,059	0,689	0,163	-0,368	-0,647	0,093
SATURATED FA	0,422	-0,362	0,412	0,704	0.909*	n.d.	0.818*	0,176	0,442	0,784	0,373	-0,038	0.997**	-0,642	-0,131	-0,592	-0,541	-0,451	-0,626	-0,423	-0,02	0,139	-0,44	1	-0,732	0,483	-0,652	-0,479	-0,206	-0.862*	0,78	-0,717	-0,627	-0,436	0,184	0,258	0,578
MONUNSATURATED FA	-0,646	0,3	-0,688	-0,679	-0,602	n.d.	-0,629	-0,171	-0,42	-0,583	0,226	0,298	-0,687	0,761	-0,414	0.890*	-0,021	0,209	0,125	-0,056	0,607	0,079	0,177	-0,732	1	-0.881*	-0,024	0,251	-0,231	0,376	-0.946*	0,73	0,154	0,51	-0,03	0,177	-0,391
C18:0/C18:1	0,727	-0,092	0.827*	0,694	0,389	n.d.	0,244	0,3	0,631	0,511	-0,444	-0,292	0,425	-0,405	0,634	-0.971**	0,261	-0,039	-0,15	0,244	-0,704	-0,154	-0,194	0,483	-0.881*	1	0,28	-0,242	0,45	-0,16	0,831	-0,349	0,22	-0,119	0,357	0,037	0,21
OMEGA-6 PUFA	0,226	0,098	0,165	-0,292	-0,726	n.d.	-0,524	-0,167	-0,183	-0,552	-0.882*	-0,417	-0,689	0,169	0,561	-0,152	0.902*	0,482	0,699	0,568	-0,688	-0,452	0,283	-0,652	-0,024	0,28	1	0,255	0,697	0,773	-0,137	0,3	0,703	0,061	-0,217	-0,523	-0,527
OMEGA-3 PUFA	-0,765	0,625	-0,198	-0,251	-0,154	n.d.	-0,276	0,371	-0,196	-0,141	0,177	0,653	-0,514	-0,048	0,507	0,392	-0,114	-0,023	0,677	0,744	0,169	0,66	0.973**	-0,479	0,251	-0,242	0,255	1	-0,515	0,758	-0,173	0,081	0,698	0,289	-0,241	-0,512	0,227
OMEGA-6/OMEGA-3	0,772	-0,358	0,295	-0,069	-0,515	n.d.	-0,235	-0,394	-0,014	-0,378	-0.920**	-0.845*	-0,213	0,174	0,129	-0,452	0.874*	0,463	0,121	-0,027	-0,735	-0.890*	-0,475	-0,206	-0,231	0,45	0,697	-0,515	1	0,119	0,027	0,234	0,228	-0,166	-0,04	-0,175	-0,717
U.I.	-0,431	0,521	-0,185	-0,502	-0,703	n.d.	-0,646	0,099	-0,316	-0,582	-0,424	0,182	-0.893*	0,311	0,528	0,317	0,501	0,394	0.832*	0,778	-0,175	0,079	0,729	-0.862*	0,376	-0,16	0,773	0,758	0,119	1	-0,392	0,422	0,828	0,342	-0,211	-0,487	-0,296
CYP1B1	0,483	-0,034	0,599	0,728	0,78	n.d.	0,706	0,456	0,47	0,758	-0,074	-0,016	0,736	-0,741	0,418	-0.895*	-0,238	-0,246	-0,173	0,181	-0,354	0,169	-0,18	0,78	-0.946*	0,831	-0,137	-0,173	0,027	-0,392	1	-0,76	-0,087	-0,344	0,166	-0,069	0,657
HMOX	-0,175	0,248	-0,073	-0,262	-0,645	n.d.	-0.953*	-0,107	0,16	-0,388	-0,137	0,075	-0,71	0.944*	0,069	0,443	0,379	0,251	-0,078	0,037	0,131	-0,085	0,059	-0,717	0,73	-0,349	0,3	0,081	0,234	0,422	-0,76	1	0,479	0,766	0,43	0,461	-0,641
IL1beta	-0,229	0,732	0,136	-0,168	-0,487	n.d.	-0,646	0,501	0,174	-0,297	-0,531	0,343	-0,676	0,475	0,796	-0,09	0,413	0,811	0,48	0,875	-0,186	0,241	0,689	-0,627	0,154	0,22	0,703	0,698	0,228	0,828	-0,087	0,479	1	0,662	0,332	-0,066	-0,068
MIP2	-0,355	0,726	-0,035	-0,053	-0,223	n.d.	-0,687	0,551	0,417	-0,023	0,127	0,607	-0,447	0.880*	0,252	0,162	-0,074	0,421	-0,192	0,374	0,386	0,512	0,163	-0,436	0,51	-0,119	0,061	0,289	-0,166	0,342	-0,344	0,766	0,662	1	0,768	0,634	-0,018
MPO	0,18	0,393	0,46	0,585	0,348	n.d.	-0,29	0,583	0.880*	0,605	0,2	0,376	0,16	0,501	0,28	-0,378	-0,216	-0,125	-0,663	0,067	0,165	0,408	-0,368	0,184	-0,03	0,357	-0,217	-0,241	-0,04	-0,211	0,166	0,43	0,332	0,768	1	0.881*	0,173
miR-21	0,139	0,12	0,247	0,488	0,376	n.d.	-0,218	0,286	0,728	0,568	0,492	0,276	0,267	0,542	-0,184	-0,102	-0,369	-0,42	-0.879*	-0,366	0,382	0,304	-0,647	0,258	0,177	0,037	-0,523	-0,512	-0,175	-0,487	-0,069	0,461	-0,066	0,634	0.881*	1	0,046
miR-155	-0,295	0,478	-0,117	0,215	0,726	n.d.	0,701	0,78	0,114	0,503	0,528	0,679	0,569	-0,378	-0,001	-0,322	-0,801	0,1	-0,124	0,298	0,431	0,805	0,093	0,578	-0,391	0,21	-0,527	0,227	-0,717	-0,296	0,657	-0,641	-0,068	-0,018	0,173	0,046	1

* p<0.05 ** p<0.01

LIVER	PROTEINS	DNA	GLYCOLIPIDS	CHOLESTEROL	PHOSPHOLIPIDS	CARDIOLIPIN	PE0	PI	PS	PC	SM	TBARS	C16:0	C16:0	C18:0	C18:1	C18:2	C18:3	C20:3	C20:4	C20:5	C22:5	C22:6	SATURATED FA	MONUNSATURATED FA	C18:0/C18:1	OMEGA6 PUFA	OMEGA3 PUFA	OMEGA6/OMEGA3	U.I.
PROTEINS	1	0.946**	-0,468	0,083	-0,374	0,759	-0,092	-0,092	0,388	-0,472	-0,075	-0,026	0,366	0,016	0,179	-0,201	-0,054	-0,51	-0,789	-0,098	0.965**	0,254	0,114	0,332	-0,182	0,246	-0,633	0,271	-0,352	-0,069
DNA	0.946**	1	-0,368	-0,043	-0,525	0,714	-0,287	-0,287	0,449	-0,611	-0,153	0,092	0,257	0,308	-0,139	0,064	0,179	-0,657	-0,791	-0,338	0.912*	0,427	-0,179	0,097	0,087	-0,04	-0,453	-0,009	-0,086	-0,344
GLYCOLIPIDs	-0,468	-0,368	1	-0,76	0,427	-0,179	0,178	0,178	-0,35	0,401	0,442	0,063	-0,792	0,341	-0,417	0,599	0,579	0,271	0,28	-0,493	-0,646	-0,547	-0,302	-0,732	0,578	-0,597	0,765	-0,456	0,54	-0,325
CHOLESTEROL	0,083	-0,043	-0,76	1	-0,183	0,249	0,194	0,194	-0,252	-0,002	-0,741	-0,587	0,685	-0,579	0,588	-0,698	-0,727	0,373	-0,112	0,694	0,256	0,364	0,492	0,75	-0,69	0,709	-0,716	0,629	-0,666	0,569
PHOSPHOLIPIDS	-0,374	-0,525	0,427	-0,183	1	-0,191	0.856*	0.856*	-0,074	0.963**	0,55	0,048	0,086	-0,604	0,385	-0,415	-0,449	0,585	0,681	0,52	-0,377	-0.937**	0,639	0,254	-0,434	0,355	-0,007	0,462	-0,383	0,651
CARDIOLIPIN	0,759	0,714	-0,179	0,249	-0,191	1	0,265	0,265	-0,184	-0,139	-0,408	-0,558	0,238	-0,112	0,255	-0,215	-0,102	0,054	-0,76	-0,026	0,696	0,111	0,227	0,287	-0,207	0,275	-0,605	0,394	-0,439	0,063
PE	-0,092	-0,287	0,178	0,194	0.856*	0,265	1	1.00**	-0,277	0.903*	0,152	-0,374	0,322	-0,786	0,63	-0,657	-0,658	0,743	0,346	0,676	-0,079	-0,779	0.836*	0,534	-0,672	0,629	-0,403	0,762	-0,707	0,809
PI	-0,092	-0,287	0,178	0,194	0.856*	0,265	10.000**	1	-0,277	0.903*	0,152	-0,374	0,322	-0,786	0,63	-0,657	-0,658	0,743	0,346	0,676	-0,079	-0,779	0.836*	0,534	-0,672	0,629	-0,403	0,762	-0,707	0,809
PS	0,388	0,449	-0,35	-0,252	-0,074	-0,184	-0,277	-0,277	1	-0,264	0,396	0.860*	0,435	0,196	-0,274	-0,031	-0,037	-0,79	0,114	-0,037	0,488	0,147	-0,126	0,145	-0,01	-0,067	-0,172	-0,125	0,051	-0,147
PC	-0,472	-0,611	0,401	-0,002	0.963**	-0,139	0.903*	0.903*	-0,264	1	0,317	-0,156	0,117	-0,648	0,414	-0,451	-0,51	0,759	0,684	0,587	-0,452	-0.854*	0,657	0,289	-0,471	0,393	-0,043	0,505	-0,424	0,699
SM	-0,075	-0,153	0,442	-0,741	0,55	-0,408	0,152	0,152	0,396	0,317	1	0,627	-0,297	-0,032	-0,008	0,097	0,147	-0,201	0,365	-0,094	-0,18	-0,695	0,102	-0,198	0,085	-0,115	0,354	-0,089	0,142	0,038
TBARS	-0,026	0,092	0,063	-0,587	0,048	-0,558	-0,374	-0,374	0.860*	-0,156	0,627	1	0,005	0,404	-0,52	0,309	0,266	-0,713	0,383	-0,266	0,017	-0,015	-0,368	-0,263	0,319	-0,408	0,333	-0,463	0,435	-0,343
C16:0	0,366	0,257	-0,792	0,685	0,086	0,238	0,322	0,322	0,435	0,117	-0,297	0,005	1	-0,568	0,458	-0,798	-0.827*	-0,026	0,074	0,74	0,582	0,147	0,579	0.890*	-0,781	0,721	-0.870*	0,678	-0,749	0,607
C16:1	0,016	0,308	0,341	-0,579	-0,604	-0,112	-0,786	-0,786	0,196	-0,648	-0,032	0,404	-0,568	1	-0.937**	0.943**	0.923**	-0,636	-0,26	-0.942**	-0,069	0,518	-0.982**	-0.852*	0.952**	-0.946**	0,646	-0.949**	0.913*	-0.992**
C18:0	0,179	-0,139	-0,417	0,588	0,385	0,255	0,63	0,63	-0,274	0,414	-0,008	-0,52	0,458	-0.937**	1	-0.887*	-0,808	0,538	-0,043	0,811	0,211	-0,399	0.926**	0.813*	-0.896*	0.946**	-0,677	0.930**	-0.900*	0.887*
C18:1	-0,201	0,064	0,599	-0,698	-0,415	-0,215	-0,657	-0,657	-0,031	-0,451	0,097	0,309	-0,798	0.943**	-0.887*	1	0.980**	-0,412	-0,136	-0.952**	-0,337	0,286	-0.938**	-0.978**	10.000**	-0.986**	0.838*	-0.964**	0.969**	-0.942**
C18:2	-0,054	0,179	0,579	-0,727	-0,449	-0,102	-0,658	-0,658	-0,037	-0,51	0,147	0,266	-0.827*	0.923**	-0,808	0.980**	1	-0,454	-0,286	-0.986**	-0,224	0,26	-0.897*	-0.956**	0.979**	-0.940**	0,78	-0.913*	0.917*	-0.943**
C18:3	-0,51	-0,657	0,271	0,373	0,585	0,054	0,743	0,743	-0,79	0,759	-0,201	-0,713	-0,026	-0,636	0,538	-0,412	-0,454	1	0,341	0,539	-0,513	-0,517	0,578	0,259	-0,434	0,434	-0,078	0,513	-0,429	0,637
C20:3	-0,789	-0,791	0,28	-0,112	0,681	-0,76	0,346	0,346	0,114	0,684	0,365	0,383	0,074	-0,26	-0,043	-0,136	-0,286	0,341	1	0,399	-0,678	-0,478	0,19	0,027	-0,148	0,02	0,318	0,008	0,048	0,353
C20:4	-0,098	-0,338	-0,493	0,694	0,52	-0,026	0,676	0,676	-0,037	0,587	-0,094	-0,266	0,74	-0.942**	0,811	-0.952**	-0.986**	0,539	0,399	1	0,066	-0,332	0.897*	0.900*	-0.956**	0.914*	-0,67	0.880*	-0.867*	0.964**
C20:5	0.965**	0.912*	-0,646	0,256	-0,377	0,696	-0,079	-0,079	0,488	-0,452	-0,18	0,017	0,582	-0,069	0,211	-0,337	-0,224	-0,513	-0,678	0,066	1	0,343	0,179	0,489	-0,314	0,353	-0,756	0,351	-0,446	0,035
C22:5	0,254	0,427	-0,547	0,364	-0.937**	0,111	-0,779	-0,779	0,147	-0.854*	-0,695	-0,015	0,147	0,518	-0,399	0,286	0,26	-0,517	-0,478	-0,332	0,343	1	-0,577	-0,108	0,308	-0,274	-0,093	-0,391	0,3	-0,53
C22-6	0,114	-0,179	-0,302	0,492	0,639	0,227	0.836*	0.836*	-0,126	0,657	0,102	-0,368	0,579	-0.982**	0.926**	-0.938**	-0.897*	0,578	0,19	0.897*	0,179	-0,577	1	0.854*	-0.946**	0.940**	-0,691	0.971**	-0.940**	0.974**
SATURATED FA	0,332	0,097	-0,732	0,75	0,254	0,287	0,534	0,534	0,145	0,289	-0,198	-0,263	0.890*	-0.852*	0.813*	-0.978**	-0.956**	0,259	0,027	0.900*	0,489	-0,108	0.854*	1	-0.971**	0.957**	-0.918**	0.921**	-0.952**	0.853*
MONUNSATURATED FA	-0,182	0,087	0,578	-0,69	-0,434	-0,207	-0,672	-0,672	-0,01	-0,471	0,085	0,319	-0,781	0.952**	-0.896*	10.000**	0.979**	-0,434	-0,148	-0.956**	-0,314	0,308	-0.946**	-0.971**	1	-0.987**	0.825*	-0.967**	0.968**	-0.951**
C18:0/C18:1	0,246	-0,04	-0,597	0,709	0,355	0,275	0,629	0,629	-0,067	0,393	-0,115	-0,408	0,721	-0.946**	0.946**	-0.986**	-0.940**	0,434	0,02	0.914*	0,353	-0,274	0.940**	0.957**	-0.987**	1	-0.837*	0.975**	-0.975**	0.925**
OMEGA-6 PUFA	-0,633	-0,453	0,765	-0,716	-0,007	-0,605	-0,403	-0,403	-0,172	-0,043	0,354	0,333	-0.870*	0,646	-0,677	0.838*	0,78	-0,078	0,318	-0,67	-0,756	-0,093	-0,691	-0.918**	0.825*	-0.837*	1	-0.832*	0.892*	-0,63
OMEGA-3 PUFA	0,271	-0,009	-0,456	0,629	0,462	0,394	0,762	0,762	-0,125	0,505	-0,089	-0,463	0,678	-0.949**	0.930**	-0.964**	-0.913*	0,513	0,008	0.880*	0,351	-0,391	0.971**	0.921**	-0.967**	0.975**	-0.832*	1	-0.993**	0.932**
OMEGA-6/OMEGA-3	-0,352	-0,086	0,54	-0,666	-0,383	-0,439	-0,707	-0,707	0,051	-0,424	0,142	0,435	-0,749	0.913*	-0.900*	0.969**	0.917*	-0,429	0,048	-0.867*	-0,446	0,3	-0.940**	-0.952**	0.968**	-0.975**	0.892*	-0.993**	1	-0.898*
U.I.	-0,069	-0,344	-0,325	0,569	0,651	0,063	0,809	0,809	-0,147	0,699	0,038	-0,343	0,607	-0.992**	0.887*	-0.942**	-0.943**	0,637	0,353	0.964**	0,035	-0,53	0.974**	0.853*	-0.951**	0.925**	-0,63	0.932**	-0.898*	1

HEART	PROTEINS	DNA	GLYCOLIPIDS	CHOLESTEROL	PHOSPHOLIPIDS	CARDIOLIPIN	PE0	PI	PS	PC	SM	TBARS	C16:0	C16:0	C18:0	C18:1	C18:2	C18:3	C20:3	C20:4	C20:5	C22:5	C22:6	SATURATED FA	MONUNSATURATED FA	C18:0/C18:1	OMEGA6 PUFA	OMEGA3 PUFA	OMEGA6/OMEGA3	U.I.	TAG
PROTEINS	1	-0,357	-0,614	0,089	0,101	-0,262	0,033	0,35	0.862*	-0,039	0,133	0,354	-0.889*	-0.863*	0.830*	-0.868*	-0.879*	-0,303	0,663	0.920**	0,67	0.909*	0.883*	0,525	-0.878*	0.823*	-0,703	0.866*	-0.824*	,890*	-0.934**
DNA	-0,357	1	0.893*	0,603	-0,436	0,326	-0,466	-0,643	-0,321	-0,558	0,026	-0,106	0,43	0,755	-0,643	0,598	0,6	-0,033	-0,34	-0,536	0,011	-0,543	-0,569	-0,632	0,633	-0,433	0,615	-0,582	0,782	-0,547	0,547
GLYCOLIPIDS	-0,614	0.893*	1	0,367	-0,159	0,632	-0,096	-0,696	-0,538	-0,21	-0,046	-0,347	0,762	0.918**	-0.884*	0.879*	0.853*	-0,052	-0,43	-0.823*	-0,332	-0.836*	-0.860*	-0,646	0.891*	-0,785	0.815*	-0.870*	0.953**	-,844*	0.820*
CHOLESTEROL	0,089	0,603	0,367	1	-0,807	0,101	-0,617	-0,728	-0,276	-0,605	-0,598	0,297	-0,14	0,157	-0,096	0,055	-0,023	-0,252	0,337	0,051	-0,091	-0,047	0,005	-0,335	0,132	0,015	0,051	-0,058	0,226	0,024	-0,055
PHOSPHOLIPIDS	0,101	-0,436	-0,159	-0,807	1	0,345	0.882*	0,365	0,335	0.839*	0,518	-0,598	0,212	-0,159	0,08	0,018	-0,006	-0,272	0,037	-0,076	0,066	-0,003	-0,077	0,514	-0,058	-0,145	-0,087	-0,052	-0,154	-0,096	0,033
CARDIOLIPIN	-0,262	0,326	0,632	0,101	0,345	1	0,613	-0,589	-0,333	0,426	-0,283	-0,447	0,611	0,447	-0,61	0,656	0,54	-0,384	0,076	-0,555	-0,558	-0,618	-0,635	-0,219	0,627	-0,763	0,55	-0,665	0,543	-0,626	0,512
PE	0,033	-0,466	-0,096	-0,617	0.882*	0,613	1	0,135	0,103	0.897*	0,074	-0,486	0,283	-0,135	-0,058	0,146	0,072	-0,302	0,178	-0,14	-0,315	-0,139	-0,173	0,369	0,073	-0,343	0,039	-0,169	-0,091	-0,186	0,092
PI	0,35	-0,643	-0,696	-0,728	0,365	-0,589	0,135	1	0,657	0,055	0,569	0,356	-0,483	-0,538	0,485	-0,561	-0,405	0,537	-0,255	0,497	0,511	0,574	0,54	0,236	-0,62	0,564	-0,303	0,611	-0,628	0,538	-0,452
PS	0.862*	-0,321	-0,538	-0,276	0,335	-0,333	0,103	0,657	1	-0,043	0,572	0,289	-0,746	-0,703	0,683	-0,75	-0,675	-0,018	0,244	0,765	0.852*	0.817*	0,747	0,41	-0,794	0,759	-0,495	0,775	-0,712	0,756	-0,756
PC	-0,039	-0,558	-0,21	-0,605	0.839*	0,426	0.897*	0,055	-0,043	1	0,05	-0,69	0,3	-0,175	0,092	0,072	-0,042	-0,494	0,319	-0,135	-0,338	-0,113	-0,131	0,629	0,029	-0,272	-0,215	-0,144	-0,143	-0,162	0,068
SM	0,133	0,026	-0,046	-0,598	0,518	-0,283	0,074	0,569	0,572	0,05	1	-0,269	-0,028	-0,001	0,13	-0,131	-0,014	0,132	-0,401	0,034	0,718	0,18	0,074	0,211	-0,177	0,236	-0,051	0,133	-0,07	0,069	-0,023
TBARS	0,354	-0,106	-0,347	0,297	-0,598	-0,447	-0,486	0,356	0,289	-0,69	-0,269	1	-0,641	-0,361	0,243	-0,437	-0,319	0,633	-0,078	0,535	0,204	0,467	0,514	-0,417	-0,434	0,494	-0,028	0,537	-0,386	0,545	-0,462
C16:0	-0.889*	0,43	0,762	-0,14	0,212	0,611	0,283	-0,483	-0,746	0,3	-0,028	-0,641	1	0.883*	-0.877*	0.956**	0.915*	-0,017	-0,504	-0.986**	-0,644	-0.970**	-0.979**	-0,381	0.948**	-0.955**	0,731	-0.975**	0.885*	-0,987	0.968**
C16:1	-0.863*	0,755	0.918**	0,157	-0,159	0,447	-0,135	-0,538	-0,703	-0,175	-0,001	-0,361	0.883*	1	-0.944**	0.949**	0.959**	0,177	-0,661	-0.947**	-0,446	-0.936**	-0.945**	-0,68	0.959**	-0.844*	0.855*	-0.936**	0.991**	-0,938	0.958**
C18:0	0.830*	-0,643	-0.884*	-0,096	0,08	-0,61	-0,058	0,485	0,683	0,092	0,13	0,243	-0.877*	-0.944**	1	-0.975**	-0.987**	-0,265	0,654	0.930**	0,588	0.950**	0.952**	0,767	-0.964**	0.920**	-0.958**	0.938**	-0.950**	0.,938**	-0.947**
C18:1	-0.868*	0,598	0.879*	0,055	0,018	0,656	0,146	-0,561	-0,75	0,072	-0,131	-0,437	0.956**	0.949**	-0.975**	1	0.976**	0,096	-0,559	-0.981**	-0,644	-0.992**	-0.995**	-0,613	0.995**	-0.970**	0.876*	-0.991**	0.961**	-0,99	0.979**
C18:2	-0.879*	0,6	0.853*	-0,023	-0,006	0,54	0,072	-0,405	-0,675	-0,042	-0,014	-0,319	0.915*	0.959**	-0.987**	0.976**	1	0,29	-0,723	-0.961**	-0,548	-0.958**	-0.968**	-0,71	0.962**	-0.913*	0.934**	-0.946**	0.948**	-0,958	0.978**
C18:3	-0,303	-0,033	-0,052	-0,252	-0,272	-0,384	-0,302	0,537	-0,018	-0,494	0,132	0,633	-0,017	0,177	-0,265	0,096	0,29	1	-0,781	-0,087	0,005	-0,076	-0,06	-0,649	0,065	0,006	0,448	0,015	0,082	-0,038	0,178
C20:3	0,663	-0,34	-0,43	0,337	0,037	0,076	0,178	-0,255	0,244	0,319	-0,401	-0,078	-0,504	-0,661	0,654	-0,559	-0,723	-0,781	1	0,592	0,063	0,53	0,557	0,689	-0,526	0,418	-0,738	0,484	-0,57	0,544	-0,658
C20:4	0.920**	-0,536	-0.823*	0,051	-0,076	-0,555	-0,14	0,497	0,765	-0,135	0,034	0,535	-0.986**	-0.947**	0.930**	-0.981**	-0.961**	-0,087	0,592	1	0,611	0.988**	0.994**	0,513	-0.978**	0.944**	-0,804	0.986**	-0.942**	0.996**	-0.995**
C20:5	0,67	0,011	-0,332	-0,091	0,066	-0,558	-0,315	0,511	0.852*	-0,338	0,718	0,204	-0,644	-0,446	0,588	-0,644	-0,548	0,005	0,063	0,611	1	0,701	0,626	0,305	-0,653	0,765	-0,455	0,653	-0,483	0,632	-0,595
C22:5	0.909*	-0,543	-0.836*	-0,047	-0,003	-0,618	-0,139	0,574	0.817*	-0,113	0,18	0,467	-0.970**	-0.936**	0.950**	-0.992**	-0.958**	-0,076	0,53	0.988**	0,701	1	0.993**	0,564	-0.993**	0.972**	-0.826*	0.993**	-0.945**	0.992**	-0.983**
C22-6	0.883*	-0,569	-0.860*	0,005	-0,077	-0,635	-0,173	0,54	0,747	-0,131	0,074	0,514	-0.979**	-0.945**	0.952**	-0.995**	-0.968**	-0,06	0,557	0.994**	0,626	0.993**	1	0,545	-0.989**	0.966**	-0.837*	0.996**	-0.953**	0.999**	-0.988**
SATURATED FA	0,525	-0,632	-0,646	-0,335	0,514	-0,219	0,369	0,236	0,41	0,629	0,211	-0,417	-0,381	-0,68	0,767	-0,613	-0,71	-0,649	0,689	0,513	0,305	0,564	0,545	1	-0,611	0,491	-0.869*	0,516	-0,664	0,51	-0,579
MONUNSATURATED FA	-0.878*	0,633	0.891*	0,132	-0,058	0,627	0,073	-0,62	-0,794	0,029	-0,177	-0,434	0.948**	0.959**	-0.964**	0.995**	0.962**	0,065	-0,526	-0.978**	-0,653	-0.993**	-0.989**	-0,611	1	-0.957**	0.849*	-0.992**	0.973**	-0,985	0.975**
C18:0/C18:1	0.823*	-0,433	-0,785	0,015	-0,145	-0,763	-0,343	0,564	0,759	-0,272	0,236	0,494	-0.955**	-0.844*	0.920**	-0.970**	-0.913*	0,006	0,418	0.944**	0,765	0.972**	0.966**	0,491	-0.957**	1	-0,806	0.971**	-0.873*	0.965**	-0.929**
OMEGA-6 PUFA	-0,703	0,615	0.815*	0,051	-0,087	0,55	0,039	-0,303	-0,495	-0,215	-0,051	-0,028	0,731	0.855*	-0.958**	0.876*	0.934**	0,448	-0,738	-0,804	-0,455	-0.826*	-0.837*	-0.869*	0.849*	-0,806	1	-0,807	0.852*	-0,813	0.841*
OMEGA-3 PUFA	0.866*	-0,582	-0.870*	-0,058	-0,052	-0,665	-0,169	0,611	0,775	-0,144	0,133	0,537	-0.975**	-0.936**	0.938**	-0.991**	-0.946**	0,015	0,484	0.986**	0,653	0.993**	0.996**	0,516	-0.992**	0.971**	-0,807	1	-0.953**	0.996**	-0.974**
OMEGA-6/OMEGA-3	-0.824*	0,782	0.953**	0,226	-0,154	0,543	-0,091	-0,628	-0,712	-0,143	-0,07	-0,386	0.885*	0.991**	-0.950**	0.961**	0.948**	0,082	-0,57	-0.942**	-0,483	-0.945**	-0.953**	-0,664	0.973**	-0.873*	0.852*	-0.953**	1	-0,945	0.946**
U.I.	0.890*	-0,547	-0.844*	0,024	-0,096	-0,626	-0,186	0,538	0,756	-0,162	0,069	0,545	-0.987**	-0.938**	0.938**	-0.990**	-0.958**	-0,038	0,544	0.996**	0,632	0.992**	0.999**	0,51	-0.985**	0.965**	-0.813*	0.996**	-0.945**	1	-0.987**
TAG	-0,934	0,547	0.820*	-0,055	0,033	0,512	0,092	-0,452	-0,756	0,068	-0,023	-0,462	0.968**	0.958**	-0.947**	0.979**	0.978**	0,178	-0,658	-0.995**	-0,595	-0.983**	-0.988**	-0,579	0.975**	-0.929**	0.841*	-0.974**	0.946**	-0.987**	1

BRAIN	PROTEINS	DNA	GLYCOLIPIDS	CHOLESTEROL	PHOSPHOLIPIDS	CARDIOLIPIN	PE0	PI	PS	PC	SM	TBARS	C16:0	C16:0	C18:0	C18:1	C18:2	C18:3	C20:3	C20:4	C20:5	C22:5	C22:6	SATURATED FA	MONUNSATURATED FA	C18:0/C18:1	OMEGA6 PUFA	OMEGA3 PUFA	OMEGA6/OMEGA3	U.I.
PROTEINS	1	0,634	0,01	-0,283	0,221	n.d.	0,519	0.846*	0,098	0,002	-0,438	-0,49	-0,645	-0,748	-0,308	-0,146	0,781	-0,01	0,244	-0,543	0,596	-,854*	0,622	-0,537	-0,227	0,004	-0,286	0,661	-0,542	0,753
DNA	0,634	1	0,051	-0,392	-0,067	n.d.	0,456	0,676	-0,392	-0,079	-0,581	-0,52	-0,71	-0,576	-0,2	0,025	0.953**	0,333	0,385	-0,593	0,064	-0,744	0,411	-0,517	-0,034	-0,1	-0,249	0,445	-0,381	0,486
GLYCOLIPIDs	0,01	0,051	1	0,156	-0,463	n.d.	0,227	-0,121	-0,579	-0,592	-0,691	-0,178	-0,636	-0,274	-0.840*	0.954**	-0,086	0.912*	0.877*	0,526	0,073	0,357	-0,69	-0.813*	0.949**	-0.968**	0,808	-0,656	0,744	-0,535
CHOLESTEROL	-0,283	-0,392	0,156	1	0,508	n.d.	0,54	-0,01	0,495	0,335	-0,192	0.889*	0,277	0,254	-0,432	0,076	-0,367	0,032	-0,296	0,62	0,246	0,292	-0,198	-0,067	0,104	-0,208	0,438	-0,192	0,292	-0,106
PHOSPHOLIPIDS	0,221	-0,067	-0,463	0,508	1	n.d.	0,599	0,595	0.904*	0.926**	0,251	0,652	0,419	-0,285	-0,043	-0,451	0,024	-0,453	-0,579	0,149	0,719	-0,239	0,349	0,22	-0,492	0,301	-0,043	0,356	-0,258	0,402
CARDIOLIPIN	n.d.	n.d.	n.d.	n.d.	n.d.	n.d.	n.d.	n.d.	n.d.	n.d.	n.d.	n.d.	n.d.	n.d.	n.d.	n.d.	n.d.	n.d.	n.d.	n.d.	n.d.	n.d.	n.d.	n.d.	n.d.	n.d.	n.d.	n.d.	n.d.	n.d.
PE	0,519	0,456	0,227	0,54	0,599	n.d.	1	0,787	0,305	0,378	-0,617	0,327	-0,391	-0,573	-0,703	0,13	0,472	0,287	0,128	0,137	0,663	-0,425	0,184	-0,598	0,074	-0,345	0,287	0,229	-0,04	0,377
PI	0.846*	0,676	-0,121	-0,01	0,595	n.d.	0,787	1	0,327	0,446	-0,356	-0,1	-0,418	-0,806	-0,343	-0,188	0,75	-0,008	0,058	-0,335	0,735	-0,781	0,553	-0,424	-0,276	0,009	-0,148	0,593	-0,447	0,691
PS	0,098	-0,392	-0,579	0,495	0.904*	n.d.	0,305	0,327	1	0,811	0,471	0,661	0,618	-0,003	0,17	-0,608	-0,222	-0,706	-0,762	0,126	0,591	-0,123	0,391	0,448	-0,623	0,496	-0,184	0,381	-0,329	0,393
PC	0,002	-0,079	-0,592	0,335	0.926**	n.d.	0,378	0,446	0,811	1	0,48	0,626	0,593	-0,202	0,189	-0,475	-0,061	-0,48	-0,63	0,147	0,538	-0,099	0,243	0,444	-0,508	0,394	-0,082	0,239	-0,197	0,231
SM	-0,438	-0,581	-0,691	-0,192	0,251	n.d.	-0,617	-0,356	0,471	0,48	1	0,231	0.874*	0,349	0,782	-0,525	-0,549	-0,714	-0,658	0,03	-0,082	0,324	0,04	0.920**	-0,502	0,65	-0,318	-0,005	-0,128	-0,14
TBARS	-0,49	-0,52	-0,178	0.889*	0,652	n.d.	0,327	-0,1	0,661	0,626	0,231	1	0,651	0,352	-0,079	-0,147	-0,523	-0,227	-0,572	0,612	0,191	0,404	-0,185	0,336	-0,114	0,07	0,301	-0,197	0,24	-0,176
C16:0	-0,645	-0,71	-0,636	0,277	0,419	n.d.	-0,391	-0,418	0,618	0,593	0.874*	0,651	1	0,564	0,629	-0,503	-0,685	-0,675	-0.812*	0,273	-0,112	0,46	-0,048	0.912*	-0,458	0,576	-0,157	-0,093	-0,007	-0,201
C16:1	-0,748	-0,576	-0,274	0,254	-0,285	n.d.	-0,573	-0,806	-0,003	-0,202	0,349	0,352	0,564	1	0,528	-0,291	-0,541	-0,362	-0,512	0,058	-0,759	0,441	-0,083	0,606	-0,194	0,391	-0,239	-0,13	-0,003	-0,238
C18:0	-0,308	-0,2	-0.840*	-0,432	-0,043	n.d.	-0,703	-0,343	0,17	0,189	0,782	-0,079	0,629	0,528	1	-0,747	-0,129	-0,766	-0,676	-0,5	-0,494	-0,052	0,403	0.893*	-0,711	0.880*	-0,761	0,354	-0,526	0,181
C18:1	-0,146	0,025	0.954**	0,076	-0,451	n.d.	0,13	-0,188	-0,608	-0,475	-0,525	-0,147	-0,503	-0,291	-0,747	1	-0,181	0.940**	0.874*	0,619	0,037	0,503	-0.834*	-0,686	0.995**	-0.973**	0.879*	-0,804	0.867*	-0,71
C18:2	0,781	0.953**	-0,086	-0,367	0,024	n.d.	0,472	0,75	-0,222	-0,061	-0,549	-0,523	-0,685	-0,541	-0,129	-0,181	1	0,118	0,231	-0,737	0,139	-0.907*	0,642	-0,465	-0,242	0,077	-0,436	0,674	-0,603	0,717
C18:3	-0,01	0,333	0.912*	0,032	-0,453	n.d.	0,287	-0,008	-0,706	-0,48	-0,714	-0,227	-0,675	-0,362	-0,766	0.940**	0,118	1	0.904*	0,413	-0,022	0,262	-0,663	-0,796	0.927**	-0.943**	0,75	-0,627	0,703	-0,527
C20:3	0,244	0,385	0.877*	-0,296	-0,579	n.d.	0,128	0,058	-0,762	-0,63	-0,658	-0,572	-0.812*	-0,512	-0,676	0.874*	0,231	0.904*	1	0,18	0,048	0,09	-0,508	-0.828*	0.843*	-0.856*	0,574	-0,47	0,533	-0,378
C20:4	-0,543	-0,593	0,526	0,62	0,149	n.d.	0,137	-0,335	0,126	0,147	0,03	0,612	0,273	0,058	-0,5	0,619	-0,737	0,413	0,18	1	0,229	0.825*	-0.842*	-0,106	0,641	-0,616	0.899*	-0.838*	0.889*	-0,772
C20:5	0,596	0,064	0,073	0,246	0,719	n.d.	0,663	0,735	0,591	0,538	-0,082	0,191	-0,112	-0,759	-0,494	0,037	0,139	-0,022	0,048	0,229	1	-0,267	0,134	-0,326	-0,041	-0,193	0,277	0,168	-0,012	0,29
C22:5	-0.854*	-0,744	0,357	0,292	-0,239	n.d.	-0,425	-0,781	-0,123	-0,099	0,324	0,404	0,46	0,441	-0,052	0,503	-0.907*	0,262	0,09	0.825*	-0,267	1	-0.887*	0,239	0,561	-0,375	0,655	-0.909*	0.843*	-,939**
C22-6	0,622	0,411	-0,69	-0,198	0,349	n.d.	0,184	0,553	0,391	0,243	0,04	-0,185	-0,048	-0,083	0,403	-0.834*	0,642	-0,663	-0,508	-0.842*	0,134	-0.887*	1	0,185	-0.863*	0,736	-0.874*	0.998**	-0.988**	,973**
SATURATED FA	-0,537	-0,517	-0.813*	-0,067	0,22	n.d.	-0,598	-0,424	0,448	0,444	0.920**	0,336	0.912*	0,606	0.893*	-0,686	-0,465	-0,796	-0.828*	-0,106	-0,326	0,239	0,185	1	-0,641	0,798	-0,493	0,133	-0,282	-0,021
MONUNSATURATED FA	-0,227	-0,034	0.949**	0,104	-0,492	n.d.	0,074	-0,276	-0,623	-0,508	-0,502	-0,114	-0,458	-0,194	-0,711	0.995**	-0,242	0.927**	0.843*	0,641	-0,041	0,561	-0.863*	-0,641	1	-0.957**	0.876*	-0.838*	0.888*	-0,752
C18:0/C18:1	0,004	-0,1	-0.968**	-0,208	0,301	n.d.	-0,345	0,009	0,496	0,394	0,65	0,07	0,576	0,391	0.880*	-0.973**	0,077	-0.943**	-0.856*	-0,616	-0,193	-0,375	0,736	0,798	-0.957**	1	-0.893*	0,698	-0,802	0,571
OMEGA-6 PUFA	-0,286	-0,249	0,808	0,438	-0,043	n.d.	0,287	-0,148	-0,184	-0,082	-0,318	0,301	-0,157	-0,239	-0,761	0.879*	-0,436	0,75	0,574	0.899*	0,277	0,655	-0.874*	-0,493	0.876*	-0.893*	1	-0.850*	0.938**	-0,743
OMEGA-3 PUFA	0,661	0,445	-0,656	-0,192	0,356	n.d.	0,229	0,593	0,381	0,239	-0,005	-0,197	-0,093	-0,13	0,354	-0,804	0,674	-0,627	-0,47	-0.838*	0,168	-0.909*	0.998**	0,133	-0.838*	0,698	-0.850*	1	-0.980**	,983**
OMEGA-6/OMEGA-3	-0,542	-0,381	0,744	0,292	-0,258	n.d.	-0,04	-0,447	-0,329	-0,197	-0,128	0,24	-0,007	-0,003	-0,526	0.867*	-0,603	0,703	0,533	0.889*	-0,012	0.843*	-0.988**	-0,282	0.888*	-0,802	0.938**	-0.980**	1	-,928**
U.I.	0,753	0,486	-0,535	-0,106	0,402	n.d.	0,377	0,691	0,393	0,231	-0,14	-0,176	-0,201	-0,238	0,181	-0,71	0,717	-0,527	-0,378	-0,772	0,29	-0.939**	0.973**	-0,021	-0,752	0,571	-0,743	0.983**	-0.928**	1
